# *Miscanthus sinensis* dominates in Andosol through strong phosphorus acquisition that enhances water-use efficiency and nitrogen fixation

**DOI:** 10.3389/fpls.2026.1819171

**Published:** 2026-04-30

**Authors:** Zhengwei Zhang, Katsuya Yano

**Affiliations:** 1 Soil Science Laboratory, Graduate School of Agricultural and Life Sciences, The University of Tokyo, Tokyo, Japan; 2 Laboratory of Crop Science, Graduate School of Bioagricultural Sciences, Nagoya University, Nagoya, Japan

**Keywords:** Andosol, *Miscanthus sinensis*, nitrogen fixation, phosphorus acquisition, sparingly soluble phosphorus forms, water-use efficiency

## Abstract

Andosol was unsuitable for crop production without large inputs of phosphorus fertilizer due to high levels of active aluminum making phosphorus insoluble. However, *Miscanthus sinensis* has dominated Andosol for centuries, and how it sustains growth despite severe phosphorus-deficiency remains unclear. Under varied phosphorus treatments, pot experiments were conducted to investigate phosphorus acquisition in *Miscanthus sinensis*, along with its impact on water-use efficiency and nitrogen fixation. Among the species tested, only *Miscanthus sinensis* showed relatively higher phosphorus acquisition in Andosol than in vermiculite, as reflected by a marked increase in biomass production (0.96 and 0.11 g plant^-1^, respectively). This provides the first experimental evidence that *Miscanthus sinensis* possesses a superior capability to utilize phosphorus sources in Andosol. This capability was further confirmed by its effective utilization of sparingly soluble phosphorus forms (Al-P, Fe-P, Ca-P, and Ca-Phy) in a vermiculite medium, where biomass production was comparable to that under phosphorus fertilizer supply. It was further observed that when C4 monocots, including *Miscanthus sinensis*, failed to acquire sufficient phosphorus, their characteristic higher water-use efficiency compared with C3 dicots disappeared. Specifically, water-use efficiency in *Miscanthus sinensis* increased from 3.2 to 7.1 g kg^-1^ across phosphorus treatments, whereas C3 dicots showed smaller variations (radish: 1.2-2.8, soybean: 4.0-5.1, and peanut: 5.1-6.2 g kg^-1^). Regression analysis showed that phosphorus concentration explained a large proportion of the variation in water-use efficiency among C4 monocots (Chinese silver grass, bahiagrass, and guinea grass) (R^2^ = 0.67), but not in C3 dicots (R^2^ = 0.04), indicating that phosphorus status is critical for maintaining the C4 trait. Furthermore, using ^15^N-enriched fertilizer, we estimated that 3.4-15.9% of nitrogen in *Miscanthus sinensis* was derived from the atmosphere, and this contribution was also strongly dependent on phosphorus availability. Our findings suggest that phosphorus acquisition contributes to maintaining high water-use efficiency and enhancing N_2_ fixation in *Miscanthus sinensis*, thereby supporting its dominance in nutrient-poor Andosol. This study improves our understanding of plant nutritional strategies under nutrient-limited conditions.

## 1 Introduction

Andosol derived from volcanic ash is widely distributed in Japan and is now used for agricultural production. This soil has unique properties and functions such as low bulk density, high water retention capacity, and high porosity, which implies potentially a highly productive soil (Shoji et al., 1993**;** Dahlgren et al., 2004). Despite these excellent properties, Andosol has only been used in agriculture since modern times by huge amounts of phosphorus (P)-fertilizer inputs because most plants suffer from serious P-deficiency due to quite high levels of active aluminum (Al) contents in the soil (Dahlgren et al., 2004**;** Takahashi and Anwar, 2007).

The name of Andosol is derived from a Japanese word meaning “dark soil”, indicating high contents of organic matter. Stable carbon isotope ratios (δ^13^C) of this soil support that the carbon source is mainly C4 plant species (Ishizuka et al., 2014). Chinese silver grass (*Miscanthus sinensis*), which is the preferred C4 species in Andosol (Ishizuka et al., 1999**;** Yoneyama et al., 2001**;** Hiradate et al., 2004**;** Shindo et al., 2005), is assumed to have played an important role in the formation of organic matter in Andosol over hundreds or thousands of years (Howlett et al., 2022). Here, a question arises: why can Chinese silver grass continuously grow in the Andosol, which induces serious P-deficiency in most plant species? However, it remained unclear until now.

P is a crucial nutrient for plant growth, being a key component of nucleic acids, ATP, and phospholipids (Marschner, 2012). Plants acquire inorganic phosphate ions (Pi) from soils, however, most soils rapidly make Pi immobile with Ca^2+^ (in neutral to alkaline soils) or Al^3+^ and Fe^3+^ (in acidic soils) by making sparingly soluble forms. In addition, organic-P constitutes a substantial portion of total soil P, generally falling within the range of 50% to 80% (Dalai, 1977), although plants cannot use organic-P unless Pi released from it. Organic P mainly derives from plant and microbial residues, and phytate is the primary component of it (Liu et al., 2022). Phytate also forms complexes with Al^3+^, Fe^3+^, and Ca^2+^, which depends on soil pH as well (Feil, 2001**;** Gerke, 2010). In particular, metal ions complexed with P determine the stability of these forms. For example, phytate complexes generally follow the stability order Al^3+^, Fe^3+^, and Ca^2+^ (Crea et al., 2008). Therefore, different P forms can influence phosphorus-use efficiency through differences in accessibility.

Plants employ various physiological strategies to cope with P-deficiency. One of these strategies is to increase the exudation of organic anions from roots, which can solubilize sparingly soluble inorganic phosphates by chelating with metal cations (Hoffland et al., 1989**;** Zhang et al., 1997). Another strategy is to mineralize organic P forms by releasing acid phosphatases, which can hydrolyze ester bonds and release Pi (Gardner et al., 1983**;** Ohwaki and Hirata, 1992). A number of plants can also form symbiotic associations with arbuscular mycorrhizal fungi extending the root surface area to enhance P uptake (Smith and Smith, 2012**;** Smith et al., 2011). Although arbuscular mycorrhizal fungi cannot directly utilize sparingly soluble sources, the mycorrhizal formation enhances P acquisition from such P sources via an interaction between root exudates solubilizing the P sources and the hyphae capturing Pi effectively (Shibata and Yano, 2003). Moreover, plants can modulate their gene expression, enzyme activities, and metabolic pathways to enhance P acquisition and allocation within the plant (Raghothama and Karthikeyan, 2005**;** Plaxton and Tran, 2011). These adaptive responses enable plants to survive and grow under P-deficient conditions.

In P-deficient conditions, plants also enhance P usage by decreasing photosynthetic rate, ATP, and NADPH production (Plaxton and Tran, 2011). This adjustment results in reduced stomatal conductance preserving their physiological balance (Radin, 1984**;** Jacob and Lawlor, 1991**;** Zhang et al., 2019). Stomatal responses are closely related to water-use efficiency (WUE) at the individual plant-level (i.e., biomass production per transpiration) (Songsri et al., 2013; Wang et al., 2018**;** Galdon-Armero et al., 2018). Additionally, several studies indicated that WUE is significantly affected by P nutrition in C3 potato (Yi and Yano, 2022) or C4 pearl millet (Payne et al., 1992). However, there is no report to investigate the relationships between WUE and the availability of sparingly soluble sources comparing C3 and C4 species. Therefore, we have attempted to investigate the correlation between WUE and the availability of sparingly soluble sources by using several plant species including not only C3 but also C4 species (including Chinese silver grass).

In addition to P acquisition, Chinese silver grass may acquire atmospheric N_2_, which can support survival in nutrient-poor Andosol. Previous studies have demonstrated that Chinese silver grass exhibits minimal dependency on nitrogen (N) fertilizers (Schwarz et al., 1994**;** Christian et al., 1997). Subsequent studies identified N_2_-fixing bacteria in Chinese silver grass (Eckert et al., 2001**;** Miyamoto et al., 2004**;** Ye et al., 2005**;** Li et al., 2022), which can contribute to N_2_ fixation, as demonstrated by 16% of N in *Miscanthus × giganteus* being derived from atmospheric N_2_ despite non-limiting soil N (Keymer and Kent, 2014). Furthermore, P availability likely plays a crucial role in N_2_ fixation by providing the energy required for the process, as Ueda and Yano (2023) indicated that the endophytic N_2_ fixation by sweet potato strongly depends on P nutrition. Given the primary prevalence of P in sparingly soluble sources within Andosol, it must be significant to elucidate how P acquisition affects N_2_ fixation by Chinese silver grass.

Based on these considerations, we hypothesized that Chinese silver grass possesses a superior capability to acquire P and to sustain growth under P-deficient Andosol, potentially through the effective utilization of sparingly soluble P forms. We further hypothesized that the availability of P from such sources would play a key role in determining WUE and atmospheric N_2_ fixation in Chinese silver grass. To test these hypotheses, Experiment 1 examined P acquisition in Chinese silver grass under natural Andosol conditions. Experiments 2 and 3 evaluated the utilization of specific sparingly soluble P forms and their effects on water-use efficiency and on N_2_ fixation in Chinese silver grass using a vermiculite-based growth medium with minimal background P, independent of inherent soil complexity. This study improves our understanding of plant nutritional strategies under P-limited conditions, with potential implications for species persistence and ecological dominance in nutrient-poor environments.

Accordingly, this study aimed to answer the following questions:

1. Does Chinese silver grass exhibit a higher P acquisition capability in Andosol than other plants?
2. Which forms of sparingly soluble P can Chinese silver grass effectively utilize?
3. Is WUE affected by the availability of sparingly soluble P forms across various species?
4. Is the rate of N_2_ fixation affected by the availability of sparingly soluble P forms in Chinese silver grass?

## 2 Materials and methods

### 2.1 Plant materials and growth conditions (experiment 1)

Chinese silver grass (*Miscanthus sinensis* Aoba, Co., Ltd., Japan) was used. Guinea grass (*Panicum maximum* Jacq. ‘Natsukaze’), soybean (*Glycine max.* cv. ‘Fukuyutaka’), amaranth (*Amaranthus* spp. Tusrushin seeds, Co., Ltd., Japan), sorghum (*Sorghum bicolor* L. Caneko seeds, Co., Ltd., Japan), barley (*Hordeum vulgare* L. cv. ‘Shunrai’), potato (*Solanum tuberosum* L. ‘Kitaakari’), sunflower (*Helianthus annuus* L. ‘Sundance’), radish (*Raphanus sativus* L. ‘Birdland’) and bahiagrass (*Paspalum notatum* cv. ‘Pensacola’) were selected as the compared plants. Seeds were sown into trays filled with vermiculite and grown in controlled-environment chambers (LPH-410 SPC, Nippon Medical and Chemical Instruments Co., Ltd., Osaka, Japan) with the following conditions: light intensity, 400 µmol m^−2^ s^−1^; relative humidity, 60%; temperature, 30 ^°^C/25 ^°^C (day/night); and photoperiod, 14 h/10 h (day/night). Potato tubers were cut into ∼6.45 g pieces and buried in the tray and sprouted in a controlled-environment room with the following conditions: light intensity, 150 µmol m^−2^ s^−1^; relative humidity, 70%; temperature, 24 ^°^C/24^°^C (day/night); and photoperiod, 12 h/12 h (day/night). After sprouting to ∼5 cm in length, the tuber pieces were placed in the same chamber as the seedlings of the other species.

The seedlings of each species were transplanted into pots (7.8 × 9.6 cm, diameter × depth; one plant per pot) filled with 300 mL of vermiculite (an artificial growth medium with minimal background nutrient supply) or Andosol (29% sand, 55% silt, and 16% clay; pH 5.7; total C 89 g kg^-1^; total N 7.2 g kg^-1^; total P 1128 mg P kg^-1^ soil; available P 0.83 mg P kg^-1^ soil). In the two media, 0.15 g of ammonium sulfate (21.0% N) and 0.06 g of potassium chloride (49.8% K) fertilizers were uniformly applied. Additionally, 0.3 g of magnesia lime was added to each pot to maintain a favorable pH environment, provide an adequate supply of Ca and Mg, and ensure comparable conditions between vermiculite and Andosol. The application rate was determined based on preliminary experiments.

Regarding P, four treatments were applied: (1) Vermiculite without P addition; (2) Andosol without P addition; (3) Andosol supplied with aluminum phosphate (0.27 g); and (4) Andosol supplied with calcium superphosphate (0.9 g, 7.6% P). P added treatments corresponded to 0.23 g P L^-1^ of medium (all values expressed per pot; **Supplementary Table S1**). Aluminum phosphate was chosen as a sparingly soluble P source to simulate the stable P forms typically found in Andosol, while calcium superphosphate served as a soluble reference. The added P dosage was determined based on preliminary experiments to ensure measurable plant responses and to allow for a clear comparison of P acquisition from sources with different availability, thereby evaluating plant capability to utilize sparingly soluble P forms. Before transplanting, each pot received 70 mL of trace elements nutrient solution (**Supplementary Table S2**) diluted at 1:1000 with tap water, to ensure adequate trace element availability in vermiculite (Ueda and Yano, 2023).

The experiment was arranged in a completely randomized design with four P treatments and three replicates. Pots were randomly repositioned to minimize positional effects. Each plant was grown in a greenhouse at the Higashiyama Campus of Nagoya University, Nagoya, Japan. The experiment started on June 27, 2021. Guinea grass, soybean, amaranth, sorghum, barley, potato, sunflower, and radish were harvested four weeks after transplanting. Chinese silver grass and bahiagrass showed slower initial growth and were harvested eight weeks after transplanting. Plants were harvested at comparable physiological stages corresponding to the late vegetative stage, as indicated by stabilization of growth or the onset of leaf senescence (e.g., leaf yellowing or abscission), to ensure comparability among species with different growth patterns. A CO_2_ recorder (TR-76Ui, T&D Inc., Japan) was placed in the greenhouse to monitor environmental conditions at 5-min intervals. The mean temperature during the growth period was 29.2 °C, and relative humidity was 77.8%.

### 2.2 Plant materials and growth conditions (experiment 2)

Peanut (*Arachis hypogaea* L. ‘Tachimasari’), soybean (*Glycine max.* cv. ‘Fukuyutaka’), and radish (*Raphanus sativus* L. ‘Birdland’) were selected to represent C3 dicots plants, and guinea grass (*Panicum maximum* Jacq. ‘Natsukaze’), Chinese silver grass (*Miscanthus sinensis* Aoba, Co., Ltd., Japan), and bahiagrass (*Paspalum notatum* cv. ‘Pensacola’) were selected to represent C4 monocots plants. The growth conditions were the same as those described in Experiment 1, except for the P treatment conditions. In Experiment 2, only using vermiculite as the growth medium and eight P treatments were used: (1) vermiculite without P addition; vermiculite supplied with (2) aluminum phosphate (0.154 g); (3) aluminum phytate (0.145 g); (4) iron phosphate (0.282 g); (5) iron phytate (0.15 g); (6) calcium monohydrogen phosphate (0.218 g); (7) calcium phytate (0.148 g); and (8) calcium superphosphate (0.514 g, 7.6% P). We adjusted the P dosage at 0.13 g P L^-1^ (all values expressed per pot; **Supplementary Table S1**) that was used in this experiment to avoid excessive P release from calcium superphosphate in vermiculite, which may negatively affect plant growth. Phytic acid salts were prepared using the following procedure: Calcium hydroxide (7.41 g), aluminum hydroxide (7.80 g), and iron hydroxide (8.99 g) were taken in separate beakers, and 100 mL of phytic acid solution (50wt.% solution, SIGMA-ALDRICH, St. Louis, MO, USA) was added to each. After sufficient reaction, the mixture was transferred into a semi-permeable membrane bag and immersed in a water-filled tank. The water in the tank was continuously exchanged using running tap water until the pH of the mixture inside the membrane bag approached neutral. The solid material inside the membrane bag was then removed, dried, and pulverized using a mill to obtain powdered forms of various phytic acid salts.

The experiment was conducted in a completely randomized design with eight P treatments and four replicates. The experiment started on August 9, 2022. Peanut, soybean, and radish were harvested four weeks after transplanting and guinea grass was harvested six weeks after transplanting, while Chinese silver grass and bahiagrass were harvested eight weeks after transplanting. The reason for different harvest times was the same as in Experiment 1. Environmental conditions were estimated using regional meteorological data (Japan Meteorological Agency, Nagoya Station), which were comparable to onsite greenhouse records during Experiment 1. For reference, the mean temperature was 27.3 °C and relative humidity of 75.5%.

### 2.3 Plant materials and growth conditions (experiment 3)

In 2023, pot experiments were conducted to evaluate the change in N_2_ fixation of Chinese silver grass (Aoba Co., Ltd., Osaka, Japan) under different P sources. The growth conditions were the same as those described in Experiment 1, but each pot (13 × 13.5 cm, diameter × depth; 1 L capacity) was filled with vermiculite as the growth medium, a nitrogen-free substrate. To assess nitrogen fixation from atmospheric N_2_, 0.5 g of ammonium sulfate (5.222 ^15^N atom%) was supplied as the sole nitrogen source and used as a tracer to determine the ^15^N atom% values in plant tissues.

In Experiment 3, eight P treatments were used: vermiculite without P addition and seven P forms were supplied at 0.13 g P L^−1^ of medium, the same as in Experiment 2: aluminum phosphate (0.515 g), aluminum phytate (0.484 g), iron phosphate (0.941 g), iron phytate (0.5 g), calcium monohydrogen phosphate (0.727 g), calcium phytate (0.492 g), or calcium superphosphate (1.714 g, 7.6% P) (all values expressed per pot; **Supplementary Table S1**). In addition, 0.19 g of potassium chloride (49.8% K), 250 mL of trace element nutrient solution (1:1,000 dilution with tap water; **Supplementary Table S2**), and 1 g of magnesia lime per pot. The experiment was conducted in a completely randomized design with eight P treatments and four replicates. Chinese silver grass was cultivated for 100 days (starting from August 15) to allow sufficient time for long-term N_2_ fixation to be assessed. Environmental conditions were estimated using regional meteorological data (Japan Meteorological Agency, Nagoya Station). For reference, the mean temperature was 22.6 °C and relative humidity of 70.7%.

### 2.4 Irrigation management and measurement of cumulative transpiration

In Experiments 1 and 3, pots were placed in trays and irrigated via bottom watering. The water level was maintained at approximately 2 cm throughout the growth period by daily monitoring and replenishment with tap water. This water level was determined through preliminary experiments to ensure an adequate water supply without causing anaerobic conditions. In Experiment 2, plants were grown in pots without drainage holes. To determine the cumulative transpiration, the soil surface of each pot was covered with plastic film to minimize evaporation, following the method of Yi et al (2019)**;** Yi et al, 2020). Accordingly, the pots were weighed daily and replenished with tap water to maintain approximately 80% of the maximum water-holding capacity. The cumulative transpiration throughout the growth period was calculated as the sum of recorded daily water consumption. WUE was calculated based on plant biomass and cumulative transpiration.

### 2.5 Quantification of the P and N in plant material and root analysis

After harvesting, plants were dried in an oven at 80 °C until they reached a constant mass to determine their dry weight. Following a previously described method (Watanabe and Olsen, 1965), P concentration was colorimetrically determined. Dried samples were ashed at 495 °C for 2 h, extracted with 4 M HCl, and reacted with a color-substrate solution (2.5 M H_2_SO_4_:4% (NH_4_)_6_Mo_7_O_24_.4H_2_O:0.1 M C_6_H_8_O_6_:4.4 mM C_8_H_4_K_2_O_12_Sb_2_ = 10:3:6:1) before measuring absorbance at 710 nm using a UV spectrophotometer (UV-1800, Shimadzu Inc., Kyoto, Japan). To determine the N concentration and ^15^N atom% in the whole plant, samples from each organ were thoroughly mixed according to their weight ratios at the time of segregation. The mixed whole-plant samples were used to determine the total N concentration using an elemental analyzer (FLASH 2000, Thermo Fisher Scientific Inc., Waltham, MA, USA). A portion of the resulting combustion gases was analyzed by an isotopic ratio mass spectrometer (Delta Plus, Thermo Fisher Scientific Inc., Waltham, MA, USA) to measure ^15^N atom% values.

Root morphological parameters (root length and root surface area) were analyzed in a flatbed scanner (EPSON EXPRESSION 10000XL, Seiko Epson Co., Nagano, Japan) using software WinRHIZO Pro LA2400 (Regent Instruments Inc., Quebec City, QC, Canada). Immediately following harvest, roots were separated from the growth medium by washing with tap water over a fine mesh sieve to ensure the complete recovery of fine roots. The cleaned roots were placed in a water-filled transparent tray and spread to minimize overlap. Scanning was performed immediately after washing to prevent root dehydration and ensure measurement accuracy.

### 2.6 Estimation of plant N derived from fertilizer and the atmosphere

According to Yano and Sekiya (2012), the nitrogen (N) derived from fertilizer (Ndff) and atmosphere (Ndfa) in total plant N content were calculated using ^15^N atom% excess due to the high ^15^N enrichment in the applied fertilizer, following these equations:

% Ndff (Nitrogen derived from fertilizer) = (^15^N atom % excess of sample/^15^N atom % excess of fertilizer) × 100.

The ^15^N atom% excess of the sample and fertilizer was calculated by subtracting the natural abundance of ^15^N (0.366%) from the ^15^N atom% of the sample and fertilizer (5.222%).

The following formula was used to calculate %Ndfa (The percentage of N derived from the atmosphere):

% Ndfa (Nitrogen derived from atmosphere) = 100 - % Ndff.

Given that vermiculite was utilized as the medium in the experiment, apart from N derived from fertilizer, the remaining N was considered to be derived from the atmosphere. The percentage of N derived from the atmosphere (%Ndfa) can be calculated by subtracting the percentage of N derived from fertilizer (%Ndff) from 100%.

### 2.7 Acetylene reduction assay

The N_2_-fixing activity of plant material was evaluated using the acetylene reduction assay (Hardy et al., 1968). In the preliminary experiment, it was determined that the lower part of the stem in Chinese silver grass is an active site for N_2_ fixation. This finding was consistent with the results of previous research conducted on rice (Ito et al., 1980; Okamoto et al., 2025), which is another member of the Poaceae family. In Experiment 3, the lower part of the stem (5 cm) was thoroughly washed under running tap water to remove the medium, and then it was packed into a glass tube (25 mL) with 1 mL of distilled water to prevent desiccation. The tube was then sealed with a silicone lid, 10% (v/v) of the gas was replaced with acetylene, and the tube was immediately incubated for two days at 30 °C/25 °C (day/night) under a 14 h/10 h (day/night) cycle. Subsequently, the ethylene concentration in each tube was determined using a gas chromatograph (GC-4000 GL Sciences Inc., Tokyo, Japan). After measurement, the samples from each tube were dried at 80 °C and weighed to indicate the acetylene reduction activity per unit of dry weight.

### 2.8 P acquisition, total biomass increase, relative growth rate, P concentration, and water-use efficiency

P acquisition (mg plant^-1^) = Plant P content - Seed P content.

Total biomass increase (g plant^-1^) = Sampling total biomass - Transplanting total biomass.

Relative growth rate (RGR) (g g^-1^ day^-1^) = (In (Sampling total biomass) - In (Transplanting total biomass))/Growing period.

P concentration (mg P g^-1^ DW) = Plant P content/Sampling total biomass.

Water-use efficiency (WUE) (g kg^-1^) = (Sampling total biomass - Transplanting total biomass)/Water consumption.

### 2.9 Statistical analyses

Data are expressed as means ± standard deviation (SD) for all experiments. Statistical analyses were conducted separately for each species, with parameters analyzed independently to evaluate the effects of P treatments. Data normality was evaluated using the Shapiro-Wilk test (*P* > 0.05). Q-Q plots were also visually inspected. Subsequently, the homogeneity of variances was evaluated using Levene’s test. When variances were homogeneous (*P* > 0.05), data were analyzed using one-way analysis of variance (ANOVA) in SPSS Statistics 23 (SPSS Inc., Chicago, IL, USA) followed by Tukey’s test for multiple comparisons. When variances were not homogeneous (*P* < 0.05), Welch’s ANOVA was applied, followed by Dunnett’s T3 test for multiple comparisons using GraphPad Prism 10 (GraphPad Software, San Diego, CA, USA). Linear regression analyses were analyzed in Origin 9.1 (OriginLab Corporation, Northampton, MA, USA).

## 3 Results

### 3.1 Interspecific comparisons of P acquisition in vermiculite and Andosol under different P amendments (experiment 1)

**Figure 1:**
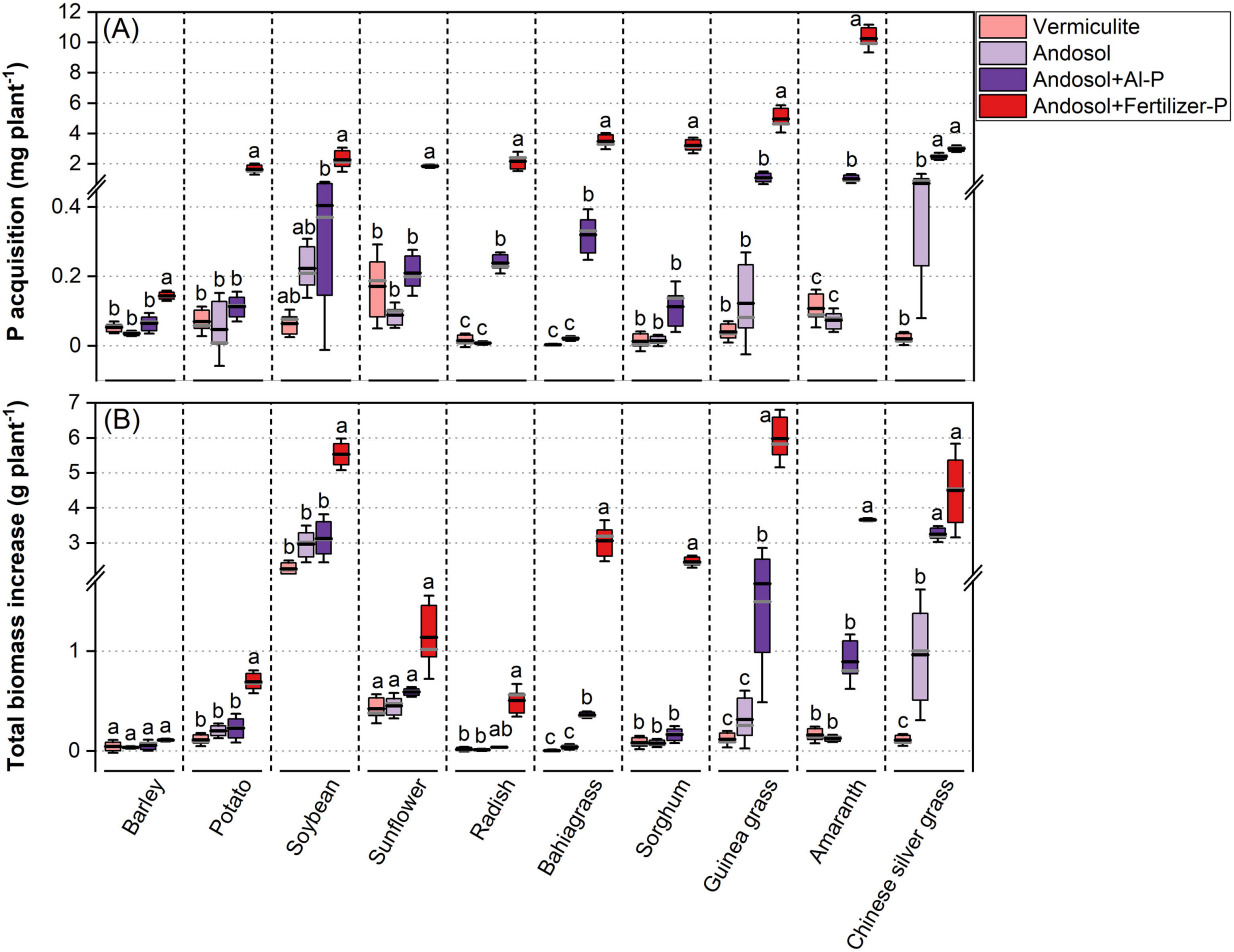
P acquisition **(A)** and total biomass increase **(B)** of barley, potato, soybean, sunflower, radish, bahiagrass, sorghum, guinea grass, amaranth, and Chinese silver grass cultivated under two phosphorus (P) levels: 0 g P kg^−1^ vermiculite (Vermiculite) or Andosol (Andosol) and 0.23 g P L^−1^ Andosol supplied as two P forms (Andosol+Al-P and Andosol+Fertilizer-P), encompassing four P treatments. Error bars indicate SD (n = 3). Columns not sharing the same letter indicate significant differences between treatments within species (*P* < 0.05), based on Tukey’s test (homogeneous variances) or Dunnett’s T3 test (non-homogeneous variances). In each box, the black line represents the mean, and the gray line indicates the median.

To compare the P acquisition of Chinese silver grass with other species in Andosol, it was compared across species across four P treatments in vermiculite and Andosol. P acquisition varied significantly among species in response to the four P treatments, with the highest values observed when soluble calcium superphosphate (Fertilizer-P) was supplied in Andosol (Andosol+Fertilizer-P) across all species (**Figure 1A**). Between the two treatments without P supply, only Chinese silver grass showed relatively higher P acquisition in Andosol (Andosol without P addition) than in vermiculite (Vermiculite without P addition) across all plants (**Figure 1A**). In addition, the P acquisition of radish, bahiagrass, amaranth, and Chinese silver grass was significantly greater in Andosol+Al-P treatment (Andosol supplied with sparingly soluble aluminum phosphate) compared with Andosol treatment (**Figure 1A**). Among these, Chinese silver grass exhibited the most pronounced increase in Andosol+Al-P treatment, which was not significantly different from that in the Andosol+Fertilizer-P treatment (**Figure 1A**). In contrast, the other plants showed no significant differences in P acquisition among the Vermiculite, Andosol, and Andosol+Al-P treatments (**Figure 1A**).

Consistent with the P acquisition patterns observed above, the total biomass increase varied among species in response to the four P treatments (**Figure 1B**). The greatest increase in total biomass occurred in the Andosol+Fertilizer-P treatment, while the Vermiculite treatment resulted in relatively less total biomass increase, except for sunflower and barley (**Figure 1B**). Among all species in the Andosol treatment, a significant increase in total biomass was only observed in Chinese silver grass, compared with the Vermiculite treatment (**Figure 1B**). In addition, bahiagrass, guinea grass, and Chinese silver grass showed a significant increase in total biomass in Andosol+Al-P treatment compared with the Andosol treatment (**Figure 1B**). In contrast, the other species showed no significant differences in total biomass increase among the Vermiculite, Andosol, and Andosol+Al-P treatments (**Figure 1B**).

### 3.2 P acquisition from sparingly soluble sources in vermiculite and its association with water-use efficiency (experiment 2)

**Figure 2:**
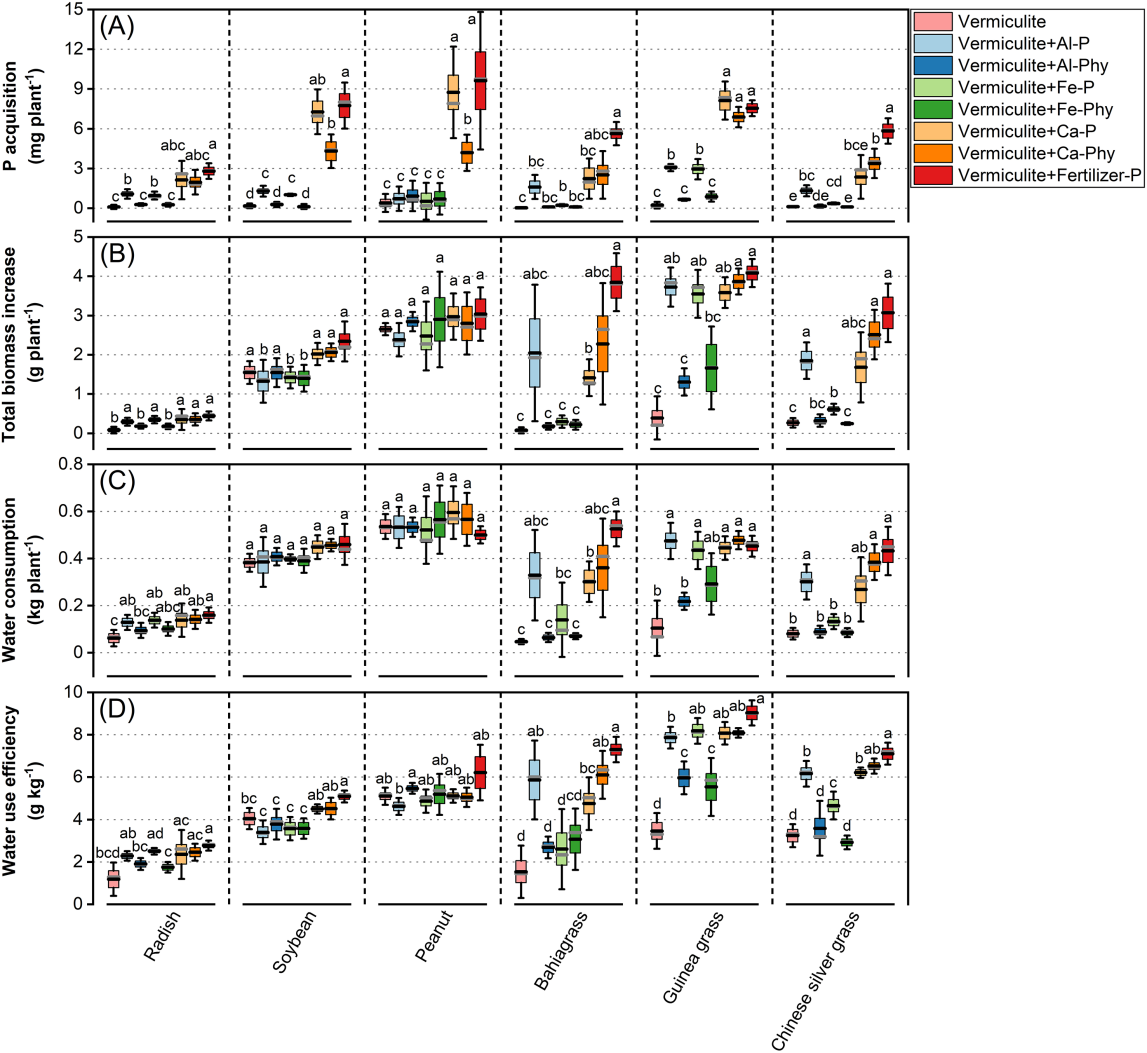
P acquisition **(A)** and total biomass increase **(B)** water consumption **(C)** water use efficiency **(D)** of radish, soybean, peanut, guinea grass, bahiagrass, and Chinese silver grass cultivated under two phosphorus (P) levels: 0 (Vermiculite) and 0.13 g P L^−1^ vermiculite supplied as seven P forms (Vermiculite+Al-P, Al-Phy, Fe-P, Fe-Phy, Ca-P, Ca-Phy, and Fertilizer-P), encompassing eight P treatments. Error bars indicate SD (n = 4). Columns not sharing the same letter indicate significant differences between treatments within species (*P* < 0.05), based on Tukey’s test (homogeneous variances) or Dunnett’s T3 test (non-homogeneous variances). In each box, the black line represents the mean, and the gray line indicates the median.

To examine which forms of sparingly soluble P can be utilized by Chinese silver grass, P acquisition was compared across eight P treatments in vermiculite (**Figure 2A**). P acquisition varied significantly among species in response to eight P treatments, with the highest levels observed when soluble calcium superphosphate (Fertilizer-P) was supplied in vermiculite (Vermiculite+Fertilizer-P) for all species, except for guinea grass (**Figure 2A**). Regarding the sparingly soluble P treatments, all species exhibited relatively greater P acquisition when calcium monohydrogen phosphate (Ca-P) or calcium phytate (Ca-Phy) was supplied in the vermiculite (Vermiculite+Ca-P or Vermiculite+Ca-Phy) compared with the other sparingly soluble P treatments (**Figure 2A**). In contrast, compared with the no P supply in vermiculite (Vermiculite), all species did not exhibit a significant increase in P acquisition when aluminum phytate (Al-Phy) or iron phytate (Fe-Phy) were supplied in vermiculite (Vermiculite+ Al-Phy or Vermiculite+Fe-Phy) (**Figure 2A**). However, radish, soybean, guinea grass, and Chinese silver grass exhibited significantly greater P acquisition when aluminum phosphate (Al-P) or iron phosphate (Fe-P) were supplied in vermiculite (Vermiculite+Al-P or Vermiculite+Fe-P) compared with the Vermiculite treatment (**Figure 2A**).

To evaluate the WUE under various P treatments, we measured both total biomass increase and water consumption across species (**Figures 2B, C**). The total biomass increase varied significantly among all species under different treatments, except for peanut (**Figure 2B**). Overall, the Vermiculite+Fertilizer-P treatment was the most effective in increasing total biomass across all species (**Figure 2B**). However, there was no significant difference between Vermiculite and Vermiculite+Fertilizer-P treatments in soybean. Under the Vermiculite+Ca-P treatment, total biomass increase in bahiagrass was significantly higher than that in Vermiculite treatment, while under the Vermiculite+Al-P and Ca-Phy treatments, it was not significantly different from that in the Vermiculite and Vermiculite+Fertilizer-P treatments. Under the Vermiculite+Al-P, Fe-P, and Ca-Phy treatments, total biomass increase in Chinese silver grass was significantly greater than in the Vermiculite treatment (**Figure 2B**). Especially, under the Vermiculite+Al-P, Ca-P, and Ca-Phy treatment, the total biomass increase in Chinese silver grass was comparable to that in the Vermiculite+Fertilizer-P treatment (**Figure 2B**). Similarly, in radish and guinea grass, total biomass increase under the Vermiculite+Al-P, Fe-P, Ca-P, and Ca-Phy treatments was comparable to that under the Vermiculite+Fertilizer-P treatment (**Figure 2B**).

Experiment 2 also demonstrates that various sparingly soluble sources have a significant effect on water consumption across species, with soybean and peanut as exceptions (**Figure 2C**). In radish, there was no significant difference in water consumption between the Vermiculite and Vermiculite+Al-Phy or Fe-Phy treatments, while all other treatments (Vermiculite+Al-P, Fe-P, Ca-P, Ca-Phy, and Fertilizer-P) showed significantly greater water consumption than that in the Vermiculite treatment (**Figure 2C**). Furthermore, significantly greater water consumption was observed in bahiagrass under Vermiculite+Ca-P treatment compared with that in the Vermiculite treatment, while the greatest water consumption was recorded in the Vermiculite+Fertilizer-P treatment (**Figure 2C**). In Chinese silver grass, water consumption was lowest in the Vermiculite treatment and there was no significant difference compared with the Vermiculite+Al-Phy, Fe-P, Fe-Phy, and Ca-P treatments (**Figure 2C**). In addition, the greatest consumption was observed in Vermiculite+Fertilizer-P, which was comparable to the Vermiculite+Al-P, Ca-P, and Ca-Phy treatments (**Figure 2C**). Notably, guinea grass exhibited no significant difference in water consumption among the Vermiculite+Al-P, Fe-P, Fe-Phy, Ca-P, Ca-Phy, and Fertilizer-P treatments (**Figure 2C**).

Consistent with the changes in biomass and water consumption, WUE exhibited significant variability among species and P treatments (**Figure 2D**). For the various P treatments, higher WUE was observed under Vermiculite+Fertilizer-P treatment and lower WUE was observed in the Vermiculite treatment across all species, except for peanut (**Figure 2D**). The WUE of radish and soybean exhibited a significant increase only under Vermiculite+Fertilizer-P treatment (2.8 and 5.1 g kg^-1^, respectively) compared with that in the Vermiculite treatment (1.2 and 4.0 g kg^-1^, respectively) (**Figure 2D**). Notably, WUE increased significantly in bahiagrass under the Vermiculite+Al-P, Ca-P, and Ca-Phy treatments (5.9, 4.8, and 6.1 g kg^-1^, respectively) compared with the Vermiculite treatment (1.5 g kg^-1^) (**Figure 2D**). Guinea grass exhibited an increased WUE across all sparingly soluble P treatments (Al-P: 7.9 g kg^-1^, Al-Phy: 6 g kg^-1^, Fe-P: 8.2 g kg^-1^, Fe-Phy: 5.5 g kg^-1^, Ca-P: 8 g kg^-1^, and Ca-Phy: 8 g kg^-1^) compared with Vermiculite treatment (3.5 g kg^-1^) (**Figure 2D**). Furthermore, Chinese silver grass showed a significant increase in WUE under Vermiculite+Al-P, Fe-P, Ca-P, and Ca-Phy treatments (6.2, 4.7, 6.2, and 6.5 g kg^-1^, respectively) compared with that in the Vermiculite treatment (3.2 g kg^-1^) (**Figure 2D**). Overall, C4 monocots (Chinese silver grass, bahiagrass, and guinea grass) showed greater variation in WUE across P treatments than C3 dicots (radish, soybean, and peanut) (**Figure 2D**).

**Figure 3:**
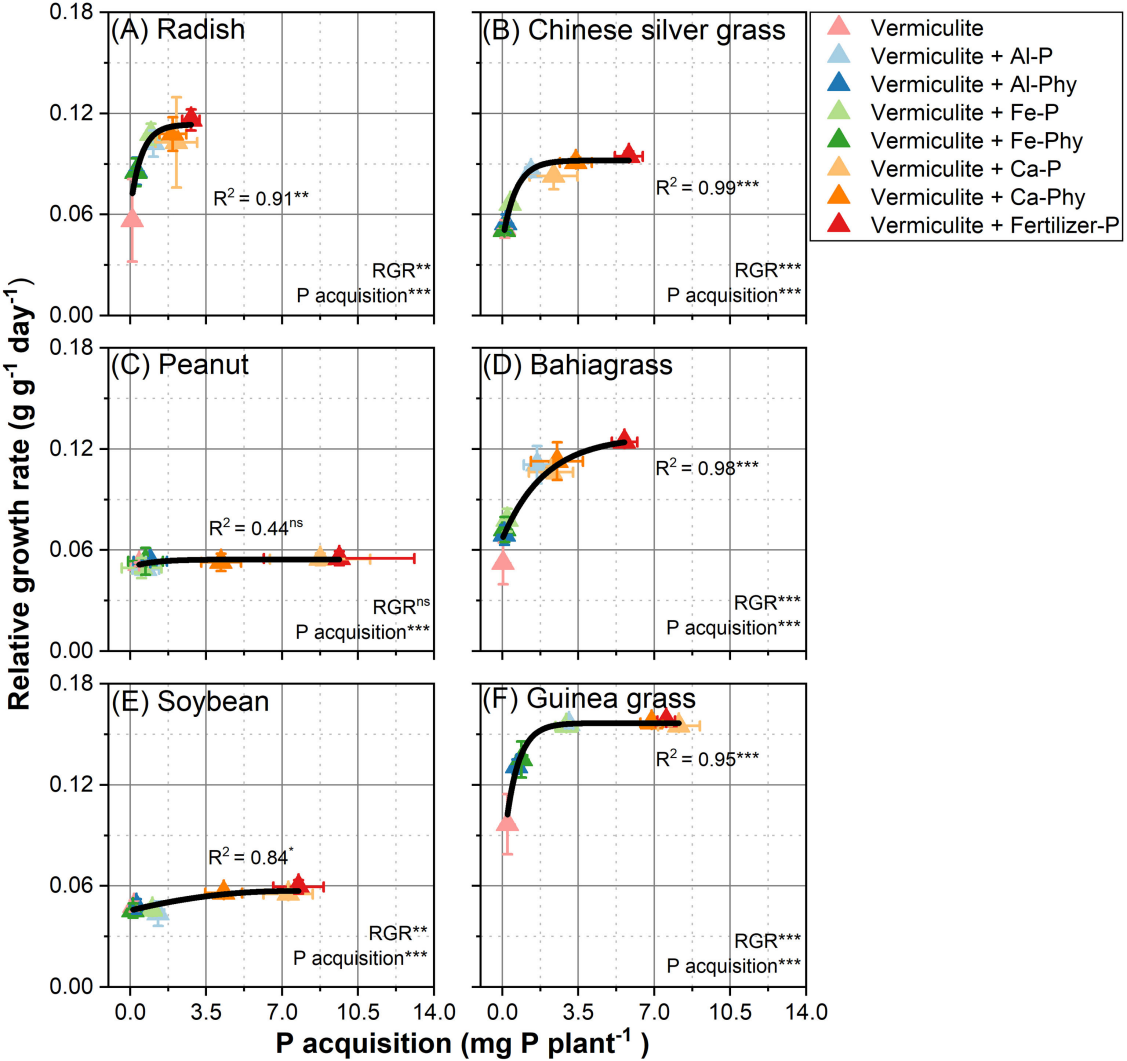
Relationships between P acquisition and relative growth rate in **(A)** Radish; **(B)** Chinese silver grass; **(C)** Peanut; **(D)** Bahiagrass; **(E)** Soybean; **(F)** Guinea grass under two phosphorus (P) levels: 0 (Vermiculite) and 0.13 g P L^−1^ vermiculite supplied as seven P forms (Vermiculite+Al-P, Al-Phy, Fe-P, Fe-Phy, Ca-P, Ca-Phy, and Fertilizer-P), encompassing eight P treatments. Error bars indicate SD (n = 4). Regressions are as follows: **(A)** *y* = 0.11 − 0.05 × 0.15^*x*^, R^2^ = 0.91; **(B)** y = 0.09 − 0.05 × 0.2^*x*^, R^2^ = 0.99; **(C)** y = 0.05 − 0.01 × 0.38^*x*^, R^2^ = 0.44; **(D)** y = 0.13 − 0.06 × 0.6^*x*^, R^2^ = 0.98; **(E)** y = 0.06 − 0.02 × 0.78^*x*^, R^2^ = 0.84; **(F)** y = 0.16 − 0.08 × 0.19^*x*^, R^2^ = 0.95. *P < 0.05, **P < 0.01, ***P < 0.001; ns, not significant.

To examine the relationship between P acquisition and relative growth rate (RGR), regression analyses were performed (**Figure 3**). The correlation between P acquisition and relative growth rate (RGR) was significantly positive for radish (**Figure 3A**), Chinese silver grass (**Figure 3B**), bahiagrass (**Figure 3D**), soybean (**Figure 3E**), guinea grass (**Figure 3F**), with *P* values less than 0.05. A positive relationship was observed between P acquisition and RGR in these species. The R^2^ values for radish (0.91), Chinese silver grass (0.99), bahiagrass (0.98), soybean (0.84), and guinea grass (0.95) show a strong fit of the regression model. In contrast, for peanut (**Figure 3C**), the correlation between P acquisition and RGR was not statistically significant (*P* > 0.05, R^2^ = 0.44).

**Figure 4:**
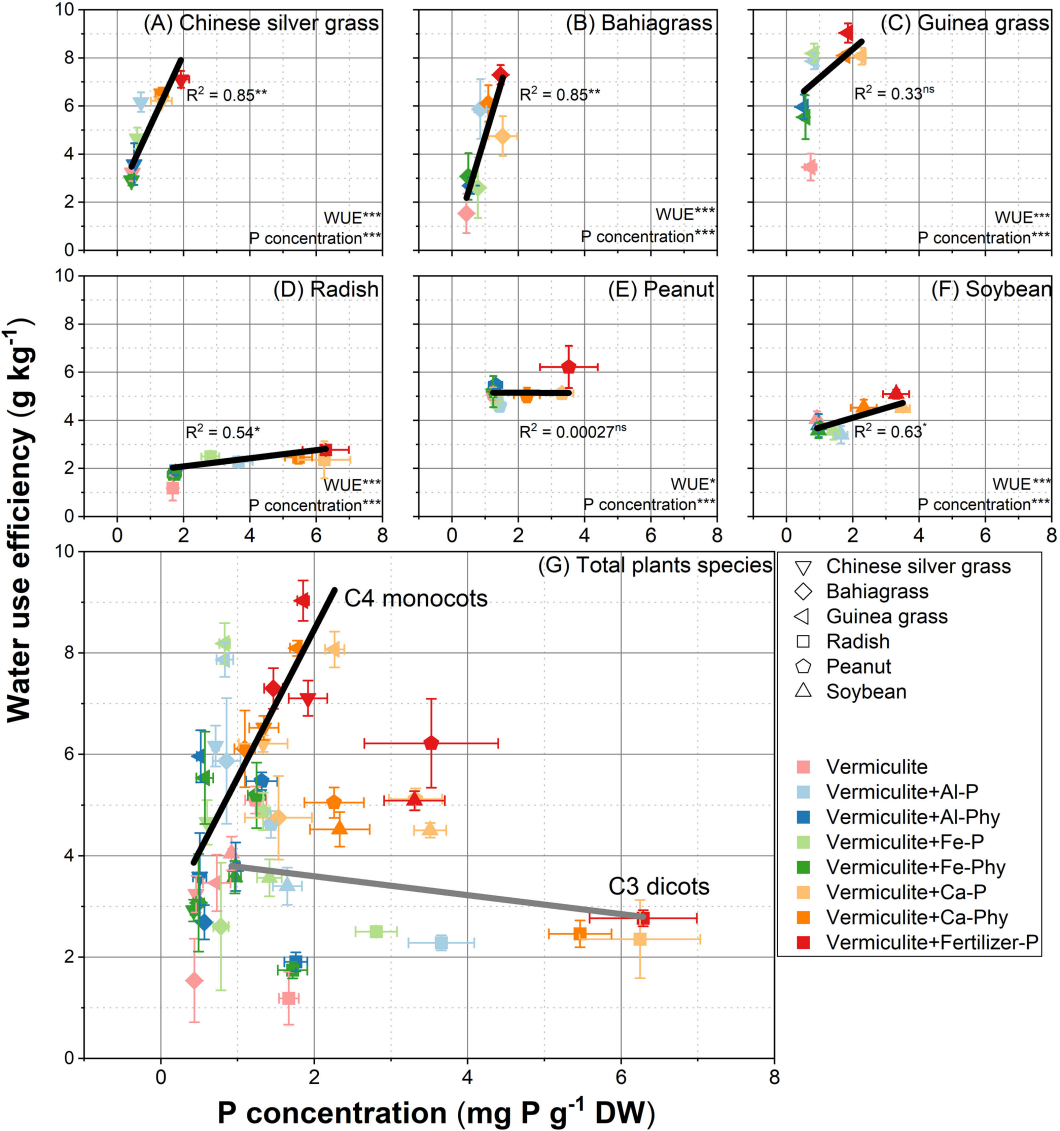
Relationships between P concentration and water use efficiency in **(A)** (Chinese silver grass); **(B)** (Bahiagrass); **(C)** (Guinea grass); **(D)** (Radish); **(E)** (Peanut); **(F)** (Soybean); **(G) (**Total species) under two phosphorus (P) levels: 0 (Vermiculite) and 0.13 g P L^−1^ vermiculite supplied as seven P forms (Vermiculite+Al-P, Al-Phy, Fe-P, Fe-Phy, Ca-P, Ca-Phy, and Fertilizer-P), encompassing eight P treatments. Error bars indicate SD (n = 4). Regressions are as follows: **(A)** *y* = 2.98*x* + 2.21, R^2^ = 0.85; **(B)** *y* = 4.56*x* + 0.19, R^2^ = 0.85; **(C)** *y* = 1.19*x* + 6.00, R^2^ = 0.33; **(D)** *y* = 0.17*x* + 1.75, R^2^ = 0.54; **(E)** *y* = −0.01*x* + 5.16, R^2^ = 2.67 × 10^-4^; **(F)** *y* = 0.41*x* + 3.29, R^2^ = 0.63; **(G)** C4 monocots: *y* = 2.93*x* + 2.61 R^2^ = 0.67; C3 dicots: *y* = 2.98*x* + 2.21, *y* = −0.19*x* + 3.97 R^2^ = 0.04. *P < 0.05, **P < 0.01, ***P < 0.001; ns, not significant.

To further examine the relationship between P and water use, linear regression was performed between P concentration and WUE (**Figure 4**). The correlation between P concentration and WUE was significantly positive for Chinese silver grass (**Figure 4A**), bahiagrass (**Figure 4B**), radish (**Figure 4D**), and soybean (**Figure 4F**), with highly significant correlations (*P* < 0.05). A positive relationship was observed between P concentration and WUE in these species. The coefficient of determination of regression was higher in Chinese silver grass (R^2^ = 0.85) and bahiagrass (R^2^ = 0.85) than in guinea grass (R^2^ = 0.33), radish (R^2^ = 0.54) and soybean (R^2^ = 0.63). Conversely, for peanut, the regression equation (y = -0.01x + 5.16) showed no significant relationship between WUE and P concentration, with a non-significant *P* value and R^2^ values of 2.7 × 10^-4^ (**Figure 4E**). Finally, the results for C4 monocots (Chinese silver grass, bahiagrass, and guinea grass) and C3 dicots (radish, soybean, and peanut) were consolidated. For C4 monocots, the R^2^ value was 0.67, with 67% of the variation in WUE explained by the variation in P concentration (**Figure 4G**). By comparison, the regression for C3 dicots (y=-0.19x + 3.97) yielded an R^2^ of 0.04, with P concentration explaining 4% of the observed variation in WUE (**Figure 4G**).

### 3.3 N_2_ fixation in Chinese silver grass as affected by P acquisition (experiment 3)

**Figure 5:**
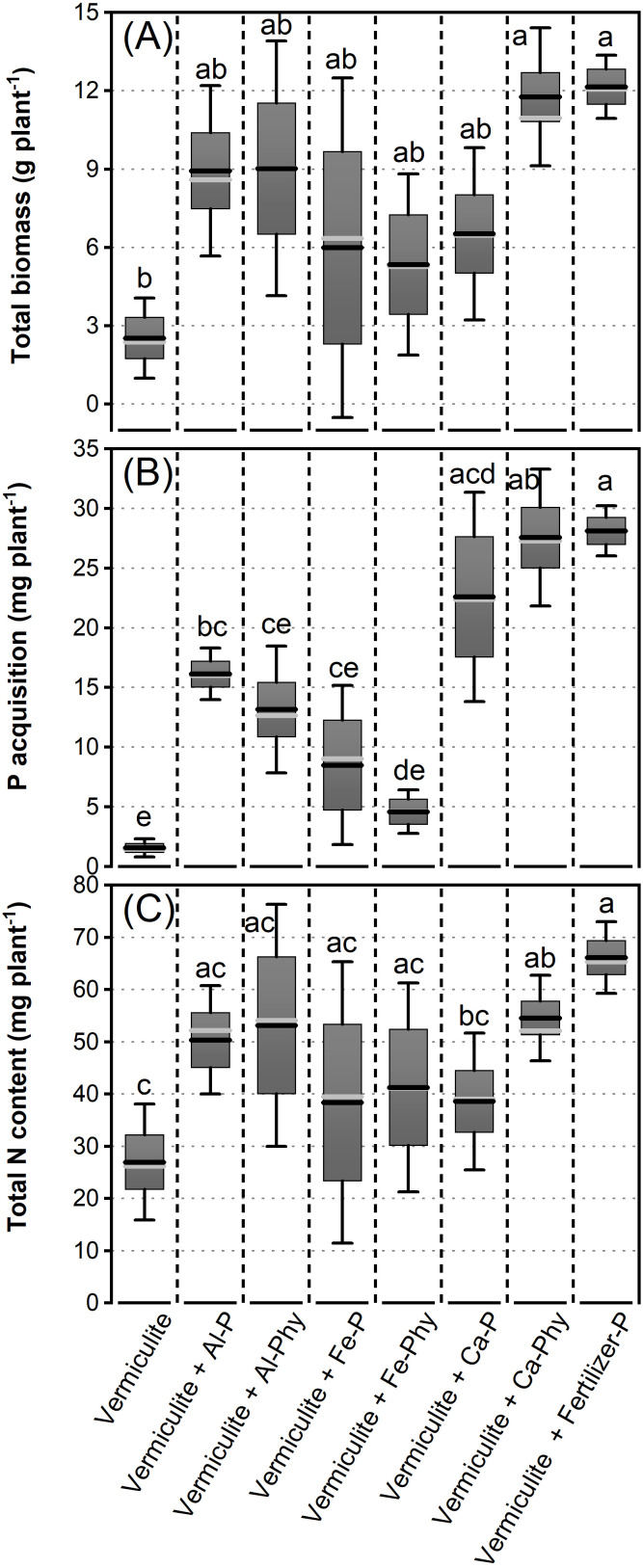
Total biomass increase **(A)** and P acquisition **(B)** and total N content **(C)** of Chinese silver grass cultivated under two phosphorus (P) levels: 0 (Vermiculite) and 0.13 g P L^−1^ vermiculite supplied as seven P forms (Vermiculite+Al-P, Al-Phy, Fe-P, Fe-Phy, Ca-P, Ca-Phy, and Fertilizer-P), encompassing eight P treatments. ^15^N-labeled ammonium sulfate (5.222 atom%) were supplied. Error bars indicate SD (n = 4); Columns not sharing the same letter indicate significant differences between treatments within species (*P* < 0.05), based on Tukey’s test (homogeneous variances) or Dunnett’s T3 test (non-homogeneous variances). In each box, the black line represents the mean, and the gray line indicates the median.

To evaluate the effect of P acquisition on the N_2_ fixation potential of Chinese silver grass, biomass, P acquisition, and total N content were measured across various P treatments (**Figures 5A–C**). As shown in **Figure 5A**, the total biomass of Chinese silver grass exhibited significant variation in response to P treatments. The greatest biomass was observed in plants under the Vermiculite+Fertilizer-P treatment, which was not significantly different from all sparingly soluble P forms but was significantly higher than that in the Vermiculite treatment (**Figure 5A**). Meanwhile, the total biomass under Vermiculite+Al-P, Al-Phy, Fe-P, Fe-Phy, and Ca-P showed modest increases, yet these did not differ significantly from the Vermiculite treatment (**Figure 5A**). The P acquisition was influenced by the P source (**Figure 5B**). P acquisition was highest in the Vermiculite+Fertilizer-P treatment, followed by the Vermiculite+Ca-Phy and Ca-P treatments, with no significant differences among these treatments (**Figure 5B**). Moderate increases in P acquisition were observed in the Vermiculite+Al-Phy, Fe-P, and Fe-Phy treatments, but these were not significantly different from the Vermiculite treatment (**Figure 5B**). Total N content varied among P treatments and followed a pattern similar to that of P acquisition (**Figure 5C**). The highest total N content was observed in the Vermiculite+Fertilizer-P treatment, which, together with the Vermiculite+Ca-Phy treatment, was significantly higher than the Vermiculite treatment (**Figure 5C**). In addition, no significant differences were observed between the other sparingly soluble P form treatments and the Vermiculite treatment (**Figure 5C**).

**Figure 6:**
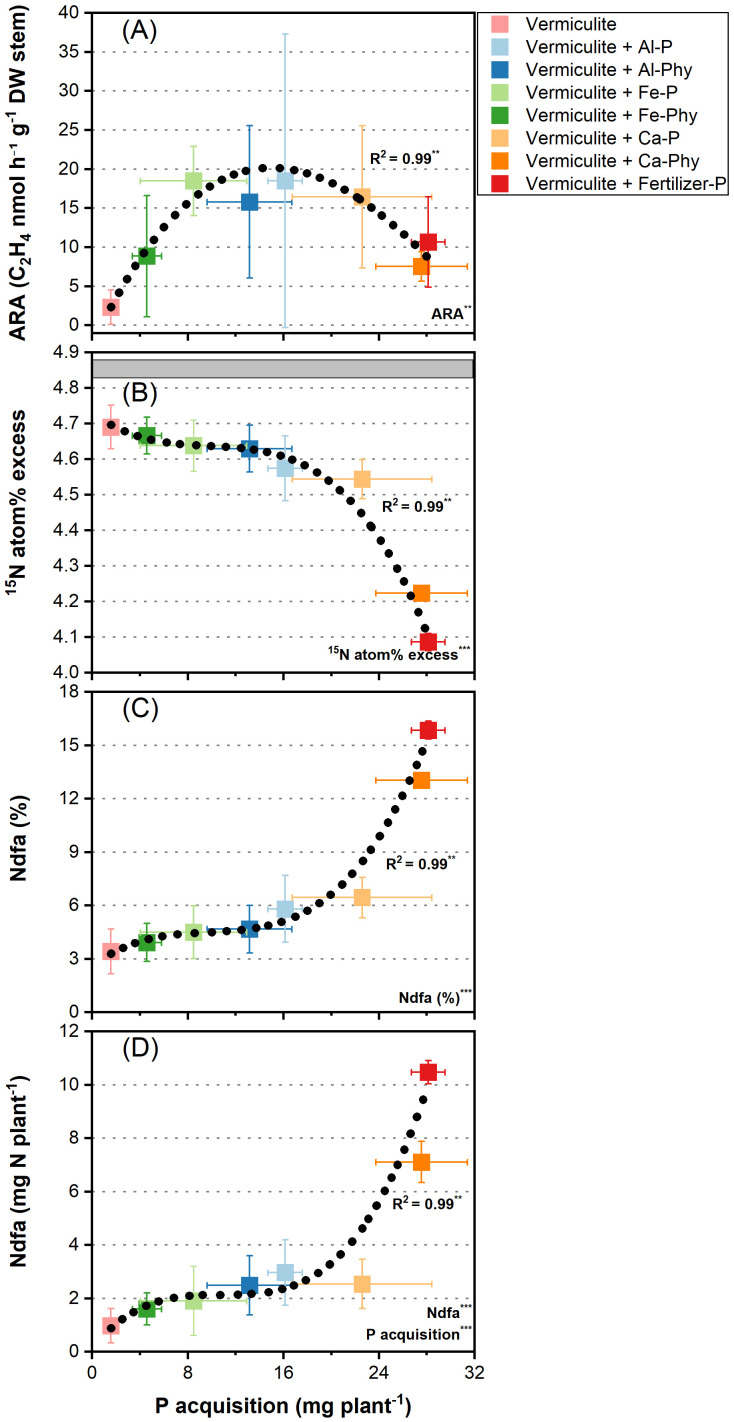
Relationships between P acquisition and ARA **(A)**, ^15^N atom% excess **(B)**, Ndfa (%) **(C)**, and Ndfa **(D)** in Chinese silver grass cultivated under two phosphorus (P) levels: 0 (Vermiculite) and 0.13 g P L^−1^ vermiculite supplied as seven P forms (Vermiculite+Al-P, Al-Phy, Fe-P, Fe-Phy, Ca-P, Ca-Phy, and Fertilizer-P), encompassing eight P treatments. ^15^N-labeled ammonium sulfate (5.222 atom%) were supplied. The gray band correspond to the range of ^15^N atom% excess of ^15^N-labeled fertilizer. Error bars indicate SD (n = 4). Regressions are as follows: **(A)**: *y* = −2.5 + 3.3*x* – 0.14*x*^2^ + 0.0012*x*^3^, R^2^ = 0.99; **(B)** *y* = 4.73 + 0.03*x* – 0.002*x*^2^ + 8.16672×10^−5^*x*^3^, R^2^ = 0.99; **(C)** *y* = 2.59 + 0.52*x* – 0.05*x*^2^ + 0.0017*x*^3^, R^2^ = 0.99; **(D)** *y* = 0.15 + 0.54*x* – 0.05*x*^2^ + 0.0015*x*^3^, R^2^ = 0.99. **P < 0.01, ***P < 0.001.

To examine the response of N_2_ fixation to P acquisition in Chinese silver grass, ARA (acetylene reduction assay), ^15^N atom% excess, and nitrogen derived from the atmosphere (%Ndfa and Ndfa) were measured across various P treatments (**Figure 6**). ARA, an indicator of N_2_-fixing activity, increased with P acquisition up to 15 mg P plant^-1^, but did not increase further at higher P acquisition (**Figure 6A**). Compared with the Vermiculite treatment, which showed the lowest ARA, all P treatments showed higher ARA (**Figure 6A**). Among the treatments, the highest ARA was observed under the Vermiculite+Al-P treatment, followed by Vermiculite+Al-Phy, Ca-P Fertilizer-P, Fe-P, Ca-Phy, and Fe-Phy (**Figure 6A**). The ^15^N atom% excess value of Chinese silver grass indicated significant variation under eight P treatments (**Figure 6B**). The gray band responds to the range of ^15^N atom% excess of the ^15^N fertilizer, serving as a reference against which plant ^15^N atom% excess can be compared (**Figure 6B**). Under Vermiculite treatment, the plants exhibited the closest ^15^N atom% excess to the ^15^N fertilizer reference (**Figure 6B**). The subsequent treatments showed a declining trend in ^15^N atom% excess, ordered as follows: Vermiculite+Fe-Phy, Fe-P, Al-Phy, Al-P, Ca-P, and Ca-Phy treatments (**Figure 6B**). The lowest ^15^N atom% excess, farthest from the ^15^N fertilizer reference, was observed in the Vermiculite+Fertilizer-P treatment (**Figure 6B**).

The %Ndfa (the percentage of nitrogen derived from the atmosphere) was positively correlated with P acquisition (**Figure 6C**). Among all P treatments, Chinese silver grass achieved the highest P acquisition and exhibited the greatest %Ndfa (15.9%) under Vermiculite+Fertilizer-P treatment (**Figure 6C**). By contrast, under the Vermiculite treatment, the lowest P acquisition and %Ndfa (3.4%) were observed (**Figure 6C**). In the remaining treatments, %Ndfa increased with elevated P acquisition, as demonstrated in the following sequence: Vermiculite+Fe-Phy (3.9%), Vermiculite+Fe-P (4.5%), Vermiculite+Al-Phy (4.7%), Vermiculite+Al-P (5.8%), Vermiculite+Ca-P (6.4%), and Vermiculite+Ca-Phy (13.0%) (**Figure 6C**). The amount of nitrogen derived from the atmosphere (Ndfa) showed a similar pattern to %Ndfa, with higher values observed at higher P acquisition (**Figure 6D**). As with %Ndfa, the highest Ndfa values (10.5 mg N plant^-1^) were observed under Vermiculite+Fertilizer-P, while the lowest (1.0 mg N plant^-1^) were observed in the Vermiculite treatment (**Figure 6D**). The remaining treatments showed increasing Ndfa values with elevated P acquisition, as demonstrated in the following sequence: Vermiculite+Fe-Phy (1.6 mg N plant^-1^), Vermiculite+Fe-P (1.9 mg N plant^-1^), Vermiculite+Al-Phy (2.5 mg N plant^-1^), Vermiculite+Ca-P (2.5 mg N plant^-1^), Vermiculite+Al-P (3.0 mg N plant^-1^), and Vermiculite+Ca-Phy (7.1 mg N plant^-1^) (**Figure 6D**).

## 4 Discussion

### 4.1 Does Chinese silver grass exhibit a higher P acquisition capability in Andosol than other plants?

Experiment 1 suggests that Chinese silver grass exhibits relatively higher P acquisition capacity in Andosol than other tested species. All species showed limited growth response in the Vermiculite treatment, which was due to the inherent absence of available P source (**Figures 1A, B**). Similarly, limited growth was observed for most species in the Andosol treatment, confirming that the P in it is largely unavailable to these species (**Figures 1A, B**). In contrast, Chinese silver grass successfully acquired P from the Andosol (**Figure 1A**), corresponding with a significant biomass increase (**Figure 1B**). As far as we know, this is the first experimental evidence that Chinese silver grass possesses a P acquisition capability that effectively enhances growth, which may provide an explanation for the prevalence of this species in Andosol where other plants fail.

Andosol is characterized by its low available P due to high contents of active Al (Saigusa et al., 1980**;** Toma and Saigusa, 1997). Therefore, AlPO_4_ (Al-P) was added to Andosol to test whether Chinese silver grass can respond to the additional Al-P. As a result, Chinese silver grass could significantly respond to the additional Al-P in terms of not only P acquisition (**Figure 1A**) but also biomass production (**Figure 1B**), suggesting that Al-P was an available P source to Chinese silver grass thus the growth was improved. However, such availability of Al-P was also found in bahiagrass, radish, and amaranth (**Figure 1A**). A previous study indicated that Andosol contains a rich variety of forms of sparingly soluble inorganic P (Pi) (e.g. Ca-, Al-, and Fe-Pi) and organic P (Po) sources (e.g. Ca-, Al-, and Fe-Po) (Otani and Ae, 1997). Because only Chinese silver grass could utilize P sources in Andosol without additional P sources (**Figure 1A**), Chinese silver grass may utilize some P sources excepting AlPO_4_, which are not readily available to bahiagrass, radish, and amaranth.

The superior P acquisition in Chinese silver grass may be associated with root exudates, particularly carboxylates released into the rhizosphere. This strategy has been observed in species native to South American acidic volcanic soils, including *Embothrium coccineum* and *Gevuina avellana* (Prance and Plana, 1998). These plants are known to exude large amounts of carboxylates in strongly P-sorbing acidic volcanic soils, facilitating P mobilization (Prance and Plana, 1998**;** Ramirez et al., 2004**;** Zúñiga-Feest et al., 2010**;** Lambers et al., 2012). Given the similar characteristics of these volcanic soils, Chinese silver grass may employ comparable rhizosphere processes to enhance P acquisition in Andosol. However, as these processes were not directly measured in this study, further investigation is required to confirm this mechanism.

### 4.2 Which forms of sparingly soluble P can Chinese silver grass effectively utilize?

In Experiment 2, to investigate which P forms can be effectively utilized by Chinese silver grass, vermiculite was used as the sole medium because of its negligible P availability as described in Experiment 1. Consequently, any observed growth response in this experiment can be attributed to the added P source. Various inorganic P forms (AlPO_4_, FePO_4_·4H_2_O, and CaHPO_4_·2H_2_O) and organic P forms (aluminum phytate, iron phytate, and calcium phytate) were individually added into the vermiculite (**Supplementary Table S1**).

As a result, however, it was difficult to identify a particular P source that is available only for Chinese silver grass, because there was no specific P source that only Chinese silver grass could respond to. There was no doubt that Chinese silver grass could effectively acquire P from Al-P in vermiculite, but the capability was also observed in radish, soybean, and guinea grass (**Figure 2A**).

However, our results indicate that P acquisition to the same P source can vary depending on the growth media used. For instance, while soybean effectively acquired P from Al-P in vermiculite (**Figure 2A**), this capability was not observed in Andosol (**Figure 1A**). This discrepancy suggests that the physiological potential of a species observed in an artificial medium (vermiculite) does not necessarily reflect its actual performance in a natural soil (Andosol). Andosol has a high amount of active Al and therefore rapidly fix P into insoluble (Dahlgren et al., 2004**;** Takahashi and Anwar, 2007), whereas vermiculite, a generally trioctahedral 2:1 clay mineral, has relatively low P-sorbing capacity (Saha and Inoue, 1997**;** Panuccio et al., 2009**;** Stylianou et al., 2007). Consequently, released P in vermiculite was likely less prone to immediate re-fixation, persisted longer in solution, and remained more available for uptake than in Andosol. Thus, we must emphasize that the results observed in Experiment 2 should be interpreted as the physiological potential inherent in these species under low P-sorbing conditions (vermiculite), rather than as a direct reflection of their performance in a natural soil (Andosol). Although Experiment 2 confirms that Chinese silver grass has the inherent physiological mechanisms to utilize sparingly soluble P, its actual dominance is more likely determined by its superior capability to sustain this P acquisition under the Andosol.

Despite this, together with previous work that evaluated P acquisition capabilities under various P sources which were supplied to sand as a medium (Pearse et al., 2008), our results suggest that the assessment of P acquisition using artificial media such as sand, vermiculite, and hydroponic systems does not necessarily reflect P acquisition from actual soil.

It is noteworthy that Chinese silver grass could utilize Fe-P in Experiment 2, and this was also observed in the other species, including radish, soybean, and guinea grass (**Figure 2A**). Furthermore, those species could effectively acquire P from Al-P (**Figure 2A**), suggesting the potential involvement of chelating activities against not only Al^3+^ but also Fe^3+^. In general, previous studies have attributed the greater P acquisition from Al-P than from Fe-P to the higher solubility of Al-P (Shane et al., 2008**;** Zhang et al., 2011). However, our results indicate that, under low P-sorption vermiculite, both Al-P and Fe-P were effectively utilized by the tested species in this study. Nevertheless, whether this Fe-P utilization observed in vermiculite persists in strongly P-sorbing Andosol remains uncertain and requires further soil-based validation.

Here, Al-Phy, Fe-Phy, and Ca-Phy were produced manually as these compounds were not available commercially, thus providing the first experimental results of Al-Phy and Fe-Phy in availabilities by plants although Ca-Phy was investigated previously (Shibata and Yano, 2003). Results showed that all plants showed no significant response to both Al-Phy and Fe-Phy, suggesting that Al^3+^ and Fe^3+^ complexes with organic phytate (Al-Phy and Fe-Phy) were more difficult for plants to utilize than those complexes with inorganic phosphate (Al-P and Fe-P) (**Figure 2A**). This result was supported by the previous report that the 6 orthophosphate moieties and 12 replaceable protons in the phytate structure render its polyanionic property and stronger ability to chelate with metal cations compared with phosphate ions (Feil, 2001; Celi and Barberis, 2005). Notably, Ca-Phy was relatively available to plants compared with Al-Phy and Fe-Phy (**Figure 2A**). These results suggest that phytate bioavailability depends on the complexed metal ions, as phytate complexes generally follow the stability order Al^3+^ > Fe^3+^ > Ca^2+^ (Crea et al., 2008). Accordingly, differences in complex stability may influence the accessibility of P from phytate complexes, thereby affecting P acquisition by plants.

Based on the results in Experiment 2, Ca-P was readily available, especially for all the dicots (radish, soybean, and peanut), which was equivalent to Fertilizer-P, compared with monocots (bahiagrass and Chinese silver grass) except guinea grass (**Figure 2A**). Compared with Ca-P, Ca-Phy was less available, particularly in soybean and peanut. In contrast, the availability of both Ca-Phy and Ca-P was similarly low in the monocots (**Figure 2A**). A previous study observed a similar result showing that P was generally more available to dicots (tomato, cabbage, and cucumber) than to monocots (rye, barley, oats, and wheat) (Deist et al., 1971). This may be contributed to the fact that dicots with higher root cation exchange capacity values absorb and retain multivalent cations, such as Ca^2+^, more efficiently than monocots (Rengel et al., 2022). Thus, the greater ability to exchange cations may contribute to the higher apparent availability of Ca-P and enhanced P acquisition in dicots compared with monocots. Furthermore, Under Ca-P, Ca-phy, and Fertilizer-P treatments, dicots showed relatively high P acquisition per root length and area, indicating root system architecture contributes to enhanced P acquisition (**Supplementary Figure S4**). In contrast, under Al- and Fe-bound P treatments, both monocots and dicots showed relatively low P acquisition per root length and area, suggesting that rhizosphere chemical processes may play an important role (**Supplementary Figure S4**).

Notably, large-seeded legumes like soybean and peanut have higher initial P reserves (**Supplementary Table S3**), which may support early growth independently of P acquisition and could lead to some underestimation of their utilization capability of sparingly soluble sources in cross-species comparisons. Nevertheless, under P-deficient conditions, seed P contributions are likely to decrease over time, and Chinese silver grass’s relatively high P acquisition probably reflects its greater capability, given its low seed P content.

### 4.3 Is WUE affected by the availability of sparingly soluble P forms across various species?

The variation in WUE across species suggests that the availability of sparingly soluble P may play a key role in regulating plant WUE (**Figure 2D**). For example, when supply of Ca-Phy to Chinese silver grass resulted in a high WUE that was comparable to comparable to that under Fertilizer-P, whereas the supply of Al-Phy and Fe-Phy resulted in a low WUE that was similar to without P supply (**Figure 2D**). This pattern suggests that plant WUE may be sensitive to differences in P availability among P sources, reflecting a coupling between P acquisition and water use regulation.

Previous studies proposed that variation in plant WUE may be small (Tanner and Sinclair, 1983**;** Sinclair et al., 1984). However, our findings align with more recent evidence indicating that variations in WUE were more significantly affected by P nutrition compared with N and K nutrition in potato (Yi and Yano, 2022). By extending these observations across a broader range of multiple species, this study demonstrates the effect of P availability on WUE across both monocots and dicots, including radish (1.2 to 2.8 g kg^-1^), soybean (3.4 to 5.1 g kg^-1^), peanut (4.6 to 6.2 g kg^-1^), bahiagrass (1.5 to 7.3 g kg^-1^), guinea grass (3.5 to 9 g kg^-1^), Chinese silver grass (3.2 to 7.1 g kg^-1^) (**Figure 2D**).

These results could be explained by mechanisms identified in previous studies, which indicated that P availability influences WUE through its effects on stomatal regulation (Singh et al., 2000**;** Huang et al., 2017). Specifically, changes in stomatal regulation alter stomatal conductance and transpiration rates, thereby modifying the balance between carbon assimilation and water loss, which determines WUE. For example, under low P supply, higher stomatal conductance and increased transpiration have been reported in white clover and *Agonis flexuosa* and white clover, thus reducing WUE (Singh et al., 2000**;** Huang et al., 2017). Conversely, sufficient P supply can reduce stomatal conductance and mitigate transpiration rates, leading to improved WUE in white clover and douglas fir (Singh et al., 2000**;** Dosskey et al., 1993).

Our findings reveal that the inherent WUE advantage of C4 species is highly dependent on P status. In comparing different plant categories, C4 monocots (Chinese silver grass, bahiagrass, and guinea grass) exhibited greater variation in WUE affected by P availability than C3 dicots (radish, soybean, and peanut) (**Figure 2D**). Specifically, C4 monocots showed higher WUE than C3 dicots especially when supplied with high availability Fertilizer P (**Figure 2D**), which is consistent with previous studies indicating that C4 species generally have higher WUE compared with C3 species (Nayyar and Gupta, 2006; Killi et al., 2017) especially under the current lower CO_2_ condition (Igarashi et al., 2021). However, without P supply, the WUE of C4 monocots were significantly declined even lower than those of C3 dicots (**Figure 2D**). This may suggest that P deficiency leads to a disproportionate decline in the photosynthetic efficiency and stomatal control of C4 monocots, causing the loss of their inherent WUE advantage. While previous studies reported that P nutritional status affect the variation of WUE in C3 potato (Yi and Yano, 2022) or C4 pearl millet (Payne et al., 1992) separately, our study demonstrates for the first time that the WUE gap between these two photosynthetic pathways is P-dependent.

The correlation between WUE and P nutritional status in both C3 dicots and C4 monocots was reflected in P concentration (**Figure 4**). The positive correlation between enhanced WUE and elevated P concentration was stronger in C4 monocots (**Figures 4A–C**) than in C3 dicots (**Figures 4D–F**). This result suggests that P concentration may be a key factor in determining the WUE of C4 monocots, implying that the plant’s capability to maintain adequate internal P levels is crucial for its water-use strategy. Therefore, efficient P acquisition from sparingly soluble sources is not only a means of nutrient uptake but also a fundamental mechanism that may enable plants to maintain water economy under environmental stress.

The relative growth rate (RGR) of soybean and peanut changed only slightly in response to increased P acquisition compared with radish and C4 monocots (**Figure 3**). This would be due to higher P content in their large seeds of soybean and peanut (**Supplementary Table S3**), which allowed to meet their P requirements independently to P acquisition from the soil during the growth period. Thus, both plants should be excluded to consider the relationship between P acquisition and WUE. However, C3 dicots radish showed apparent greater variation in RGR (**Figure 3A**) with increased P acquisition but maintained a relatively stable WUE range (1.2 to 2.8 g kg^-1^). Such a relatively stable variation range 3 to 6 kg^-1^ in C3 dicots potato according to P nutrition was also observed in the previous study (Yi and Yano, 2022). In contrast, the WUE of C4 monocots in this study, including bahiagrass (1.5 to 7.3 g kg^-1^), guinea grass (3.5 to 9 g kg^-1^), Chinese silver grass (3.2 to 7.1 g kg^-1^), revealed remarkably greater variations according to P concentration (**Figures 4A–C**). Therefore, when evaluating the relationship between P nutritional status and WUE, the effect of inherent P reserves in the seeds should be considered, which in turn, may affect the plant’s WUE.

### 4.4 How is the N_2_ fixation affected by the availability of sparingly soluble P forms in Chinese silver grass?

Chinese silver grass maintained N accumulation even when P availability from sparingly soluble sources was limited. Notably, although P acquisition from Al-P, Al-Phy, Fe-P, and Fe-Phy was significantly lower than that from fertilizer-P, total N content did not differ among these treatments (**Figures 5B, C**). This suggests that reduced P acquisition did not translate into lower N accumulation. This observation may be attributed to the plant’s adaptive mechanisms that optimize P utilization (Raghothama and Karthikeyan, 2005**;** Plaxton and Tran, 2011), which may support N assimilation processes despite low P available in Chinese silver grass. Therefore, the capability of Chinese silver grass to maintain N content under limited P availability underscores its potential for sustain growth in P-deficient environments.

The lowest ARA was observed in the no P supply treatment, and other P treatments generally led to higher ARA, indicating that P sources affect N_2_-fixing activity in Chinese silver grass (**Figure 6A**). Especially under Al-P treatment, ARA attained a saturation at a maximum rate of 18.49 C_2_H_4_ nmol h^-1^ g^-1^ DW stem when P acquisition reached approximately 15 mg P plant^-1^, suggesting that P deficiency constrained ARA unless alleviated by adequate P conditions as previously suggested (Vadez et al., 1997). However, the results of this study demonstrate a decline in ARA beyond this P acquisition threshold (>15 mg P plant^-1^) in Chinese silver gras, challenging the conventional view that sufficient P availability uniformly sustains or enhances N_2_-fixing activity (**Figure 6A**).

The lower ^15^N atom% excess values in Chinese silver grass at each P treatment in comparison with those in the ^15^N fertilizer were attributed to the accumulation of N derived from the atmosphere (Ndfa), with variations in Ndfa reflecting differences in N_2_-fixing capability (**Figures 6C, D**). However, the steady increase in %Ndfa and Ndfa with increased P acquisition does not correspond to the pattern observed in ARA, suggesting that short‐term measurements of N_2_-fixing activity may not reliably capture the cumulative atmospheric N contribution to total plant N (**Figures 6A, C, D**). Therefore, both short-term activity (ARA) and cumulative indicators (%Ndfa, Ndfa) indicators should be considered when evaluating N_2_ fixation.

While the Fertilizer-P treatment yielded a %Ndfa value of 15.9%, aligning closely with the previously reported value of 16% in *Miscanthus × giganteus* (Keymer and Kent, 2014), our study further expanded the scope by quantifying %Ndfa values under various P conditions. Specifically, the %Ndfa value was firstly observed under the treatment without P addition (3.4%), and varying values were also recorded under treatments with sparingly soluble sources, including Fe-Phy (3.9%), Fe-P (4.5%), Al-Phy (4.7%), Al-P (5.8%), Ca-P (6.4%), and Ca-Phy (13.0%). The present findings suggest that the availability of P sources influence the extent of N_2_ fixation in Chinese silver grass. However, minor non-biological N incorporation during the long cultivation (100 days) cannot be excluded, and thus the results of %Ndfa estimates should be regarded as conservative.

Our study shows a P-dependent response of N_2_ fixation in Chinese silver grass under consistent N input conditions. This dependence can be explained by the high energy demand of nitrogenase, as N_2_ reduction is an inherently energy-expensive process, requiring approximately 16 mol ATP per mol N_2_ fixed (Marschner, 1995), and P is essential for ATP production and energy metabolism. While legumes require substantial P for nodule development and function (Marschner, 1995), similar P-dependent N_2_ fixation has also been observed in sweet potato, where endophytic N_2_ fixation strongly correlates with P supply despite the absence of specialized organs (Ueda and Yano, 2023). Given that endophytic N_2_-fixing bacteria have also been identified in Chinese silver grass (Eckert et al., 2001**;** Miyamoto et al., 2004**;** Ye et al., 2005**;** Li et al., 2022), the observed P-responsiveness is likely mediated through these microbial associations.

However, P acquisition from Ca-Phy and Ca-P was comparable (**Figure 5B**), whereas Ca-Phy resulted in a stronger N_2_ fixation in Experiment 3 (**Figures 6C, D**). A possible explanation is that Ca-P provides only inorganic P, whereas Ca-Phy can supply not only P but also inositol-derived carbon compounds, which could stimulate microbial activity associated with N_2_ fixation. Previous studies have indicated that certain host-associated microbes can utilize inositol as a carbon source to modulate microbial activity, and that diazotrophs can use specific carbon sources to enhance N_2_ fixation in rice plants (O’Banion et al., 2023**;** Okamoto et al., 2025). These observations suggest that the combined availability of P and carbon sources may influence N_2_ fixation in Chinese silver grass.

From an ecological perspective, diazotrophic plants such as *Casuarina glauca* and Proteaceae species that colonize soils with strong P-sorbing promote their N_2_ fixation through P-mobilizing capability, thereby facilitating their establishment and growth (Diem et al., 2000**;** Lambers et al., 2012). Our findings suggest that Chinese silver grass possibly also employs a similar strategy, whereby efficient P acquisition from sparingly soluble sources contributes to enhancing endophytic N_2_ fixation. Such a P-driven N strategy offers a potential explanation that Chinese silver grass maintains productivity and dominance in poor-nutrient soils. However, further studies are required to confirm these integrated mechanisms and their contribution to the plant’s dominance within native Andosol environments.

## 5 Conclusion

This study shows that Chinese silver grass possesses a capability to acquire P in P-deficient Andosol, which was not observed in the other tested species. In the subsequent experiments, using a vermiculite-based growth medium to provide a controlled growth condition with minimal P interference. Our results further suggest that Chinese silver grass can effectively utilization various sparingly soluble P forms, including Al-P, Fe-P, Ca-P, and Ca-Phy. We found that the availability of P sources played a critical role in regulating WUE, with C4 monocots showing a stronger P-dependent maintenance of WUE than C3 dicots. Furthermore, we provide quantitative evidence that P acquisition from sparingly soluble sources is essential for N_2_ fixation in Chinese silver grass, with nitrogen derived from the atmosphere ranging from 3.4% to 15.9% across P treatments.

In conclusion, our results suggest that phosphorus acquisition serves as a potential adaptive strategy for Chinese silver grass in Andosol. By acquiring P from sparingly soluble sources, Chinese silver grass not only shows a P-dependent increase in N_2_ fixation but also maintains a high WUE. The combined effects of P, N, and water provide a possible explanation for the sustained productivity and dominance of Chinese silver grass in nutrient-poor Andosol.

## Data Availability

The original contributions presented in the study are included in the article/**Supplementary Material**. Further inquiries can be directed to the corresponding authors.
