# Supplementary material for: Miscanthus sinensis dominates in Andosol through strong phosphorus acquisition that enhances water-use efficiency and nitrogen fixation
Source: Front Plant Sci. 2026 Apr 30;17:1819171. doi: 10.3389/fpls.2026.1819171 (PMC13171766; doi:10.3389/fpls.2026.1819171)
Supplement: Supplementary file 1 [file DataSheet1.docx]

**
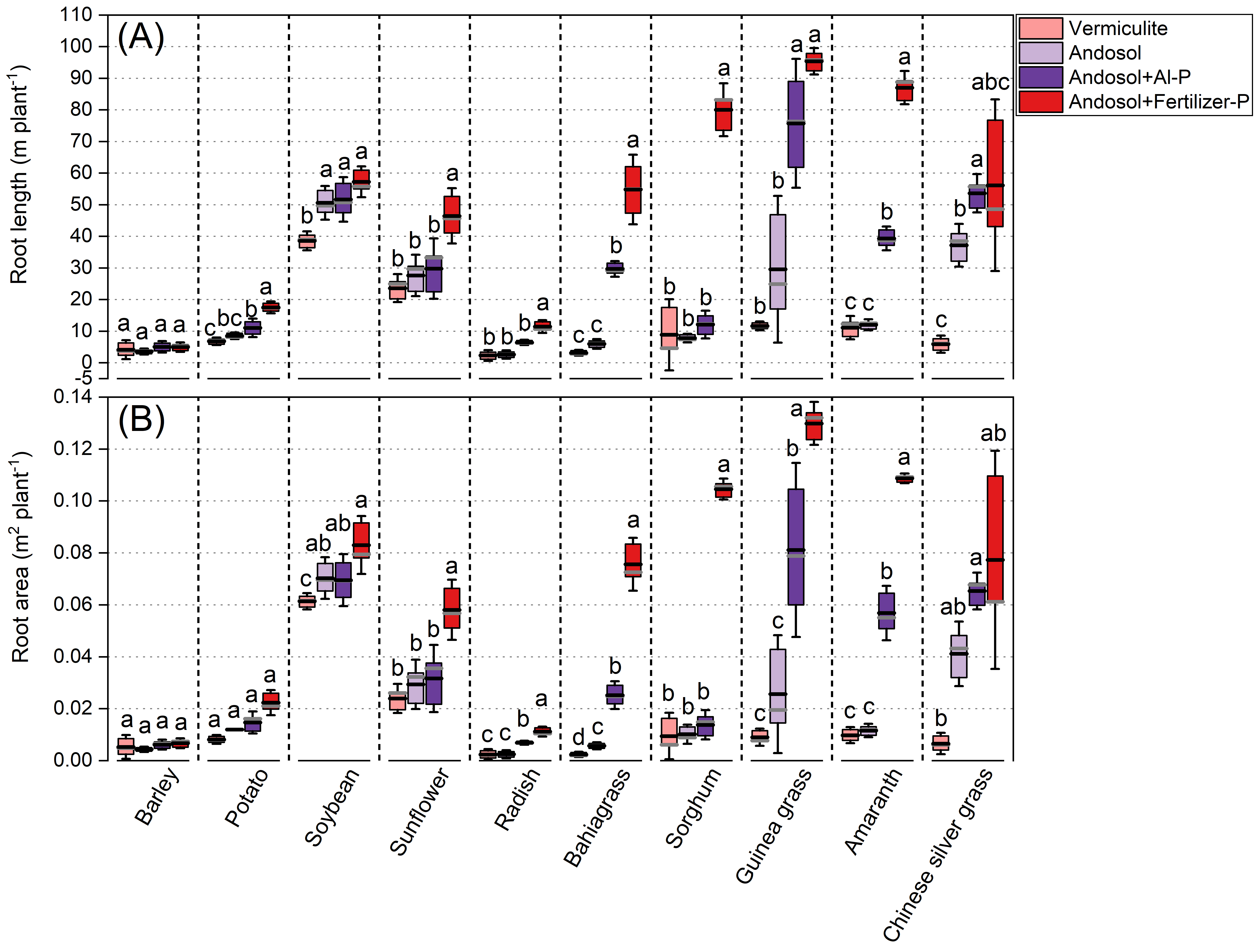
**

**Figure S1.** Root length (A) and root area (B) of barley, potato, soybean, sunflower, radish, bahiagrass, sorghum, guinea grass, amaranth, and Chinese silver grass cultivated under two phosphorus (P) levels: 0 g P kg⁻¹ vermiculite (Vermiculite) or Andosol (Andosol), and 0.23 g P L⁻¹ Andosol supplied as two P forms (Andosol+Al-P and Andosol+Fertilizer-P), encompassing four P treatments. Error bars indicate SD (n = 3). Columns not sharing the same letter indicate significant differences between treatments within species (*P* < 0.05), based on Tukey’s test (homogeneous variances) or Dunnett’s T3 test (non-homogeneous variances). In each box, the black line represents the mean, and the grey line indicates the median.

**
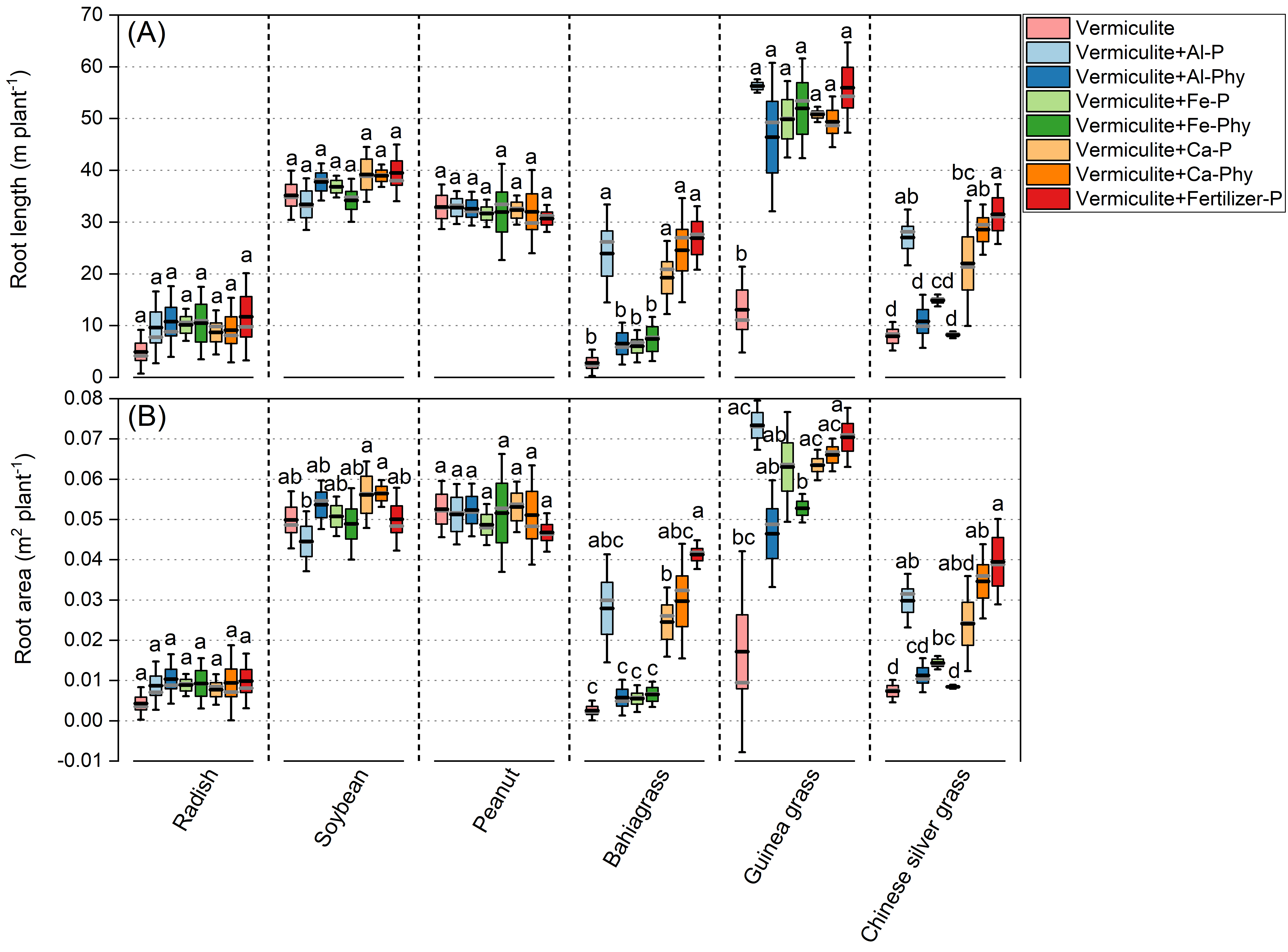
**

**Figure S2.** Root length (A) and root area (B) of radish, soybean, peanut, guinea grass, bahiagrass, and Chinese silver grass cultivated under two P levels: 0 (Vermiculite) and 0.13 g P L⁻¹ vermiculite supplied as seven P forms (Vermiculite+Al-P, Al-Phy, Fe-P, Fe-Phy, Ca-P, Ca-Phy, Fertilizer-P), encompassing eight P treatments. Error bars indicate SD (n = 4). Columns not sharing the same letter indicate significant differences between treatments within species (*P* < 0.05), based on Tukey’s test (homogeneous variances) or Dunnett’s T3 test (non-homogeneous variances). In each box, the black line represents the mean, and the grey line indicates the median.

**
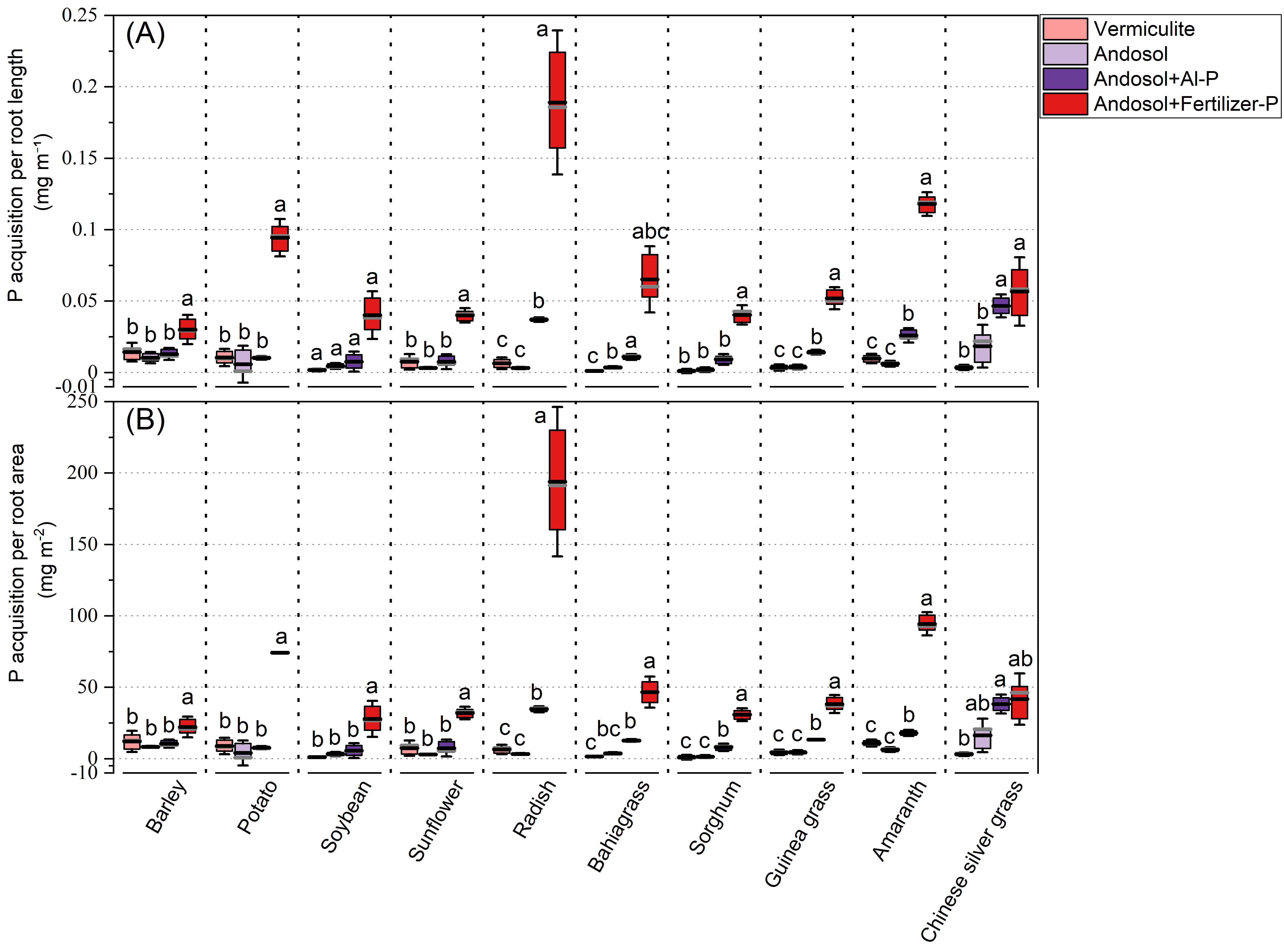
**

**Figure S3.** P acquisition per root length (A) and P acquisition per root area (B) of barley, potato, soybean, sunflower, radish, bahiagrass, sorghum, guinea grass, amaranth, and Chinese silver grass cultivated under two phosphorus (P) levels: 0 g P kg⁻¹ vermiculite (Vermiculite) or Andosol (Andosol), and 0.23 g P L⁻¹ Andosol supplied as two P forms (Andosol+Al-P and Andosol+Fertilizer-P), encompassing four P treatments. Error bars indicate SD (n = 3). Columns not sharing the same letter indicate significant differences between treatments within species (*P* < 0.05), based on Tukey’s test (homogeneous variances) or Dunnett’s T3 test (non-homogeneous variances). In each box, the black line represents the mean, and the grey line indicates the median.

**
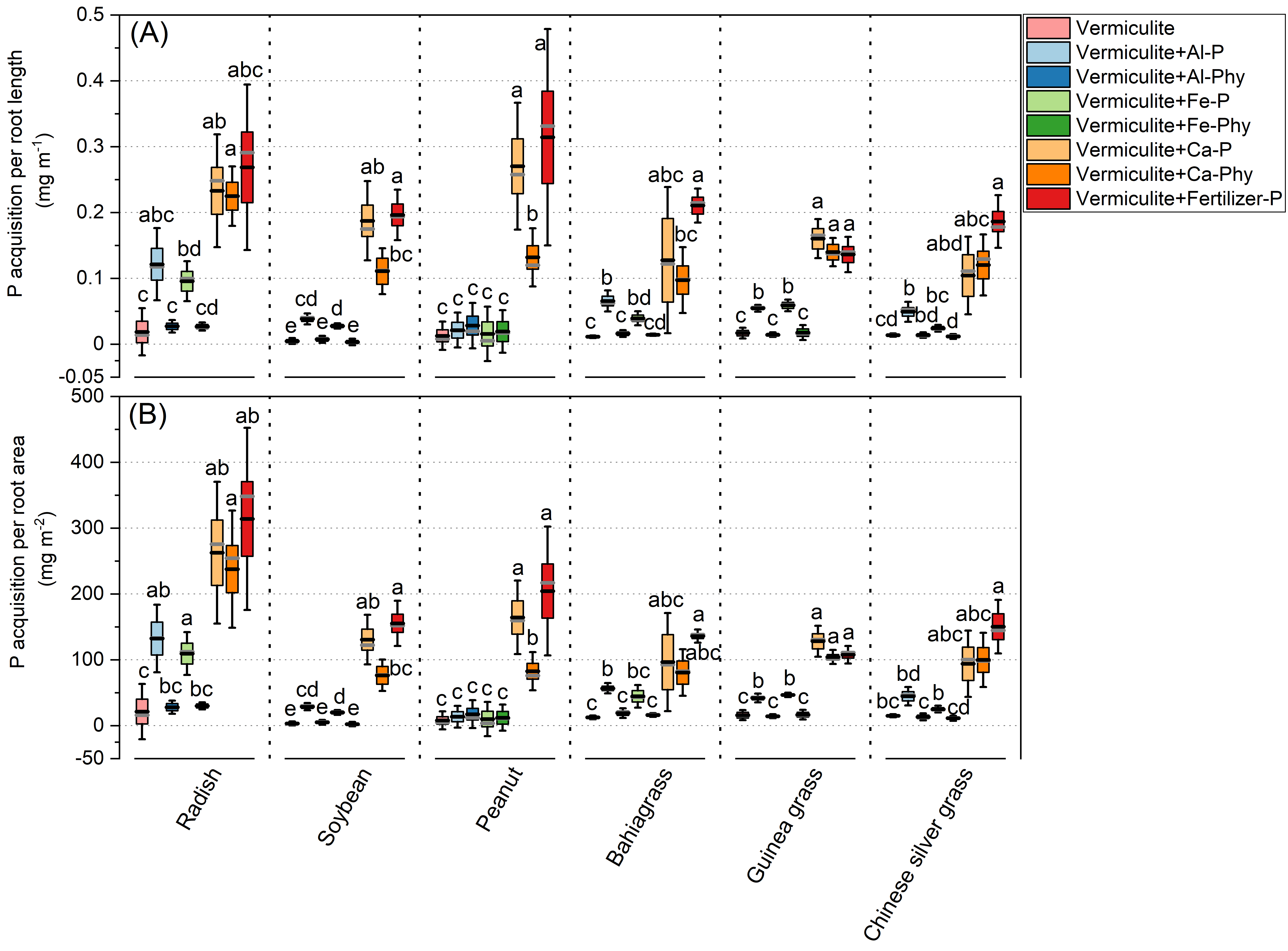
**

**Figure S4.** P acquisition per root length (A) and P acquisition per root area (B) radish, soybean, peanut, guinea grass, bahiagrass, and Chinese silver grass cultivated under two P levels: 0 (Vermiculite) and 0.13 g P L⁻¹ vermiculite supplied as seven P forms (Vermiculite+Al-P, Al-Phy, Fe-P, Fe-Phy, Ca-P, Ca-Phy, Fertilizer-P), encompassing eight P treatments. Error bars indicate SD (n = 4). Columns not sharing the same letter indicate significant differences between treatments within species (*P* < 0.05), based on Tukey’s test (homogeneous variances) or Dunnett’s T3 test (non-homogeneous variances). In each box, the black line represents the mean, and the grey line indicates the median.
