# Supplementary material for: Miscanthus sinensis dominates in Andosol through strong phosphorus acquisition that enhances water-use efficiency and nitrogen fixation
Source: Front Plant Sci. 2026 Apr 30;17:1819171. doi: 10.3389/fpls.2026.1819171 (PMC13171766; doi:10.3389/fpls.2026.1819171)
Supplement: Supplementary file 2 [file Table1.docx]

Table S1. Utilization of various P treatments

| Experiment 1 | | | |
| --- | --- | --- | --- |
| Media | Phosphate form | g pot^-1^ | P treatment |
| Vermiculite | No P applied | - | Vermiculite |
| Andosol | No P applied | - | Andosol |
| Andosol | Aluminum phosphate | 0.27 | Andosol + Al-P |
| Andosol | Calcium superphosphate | 0.9 | Andosol + Fertilizer-P |
|  |  |  |  |
| Experiment 2 | | | |
| Medium | Phosphate form | g pot^-1^ | P treatment |
| Vermiculite | No P applied | - | Vermiculite |
| Vermiculite | Aluminum phosphate | 0.154 | Vermiculite + Al-P |
| Vermiculite | Aluminum phytate | 0.145 | Vermiculite + Al-Phy |
| Vermiculite | Iron phosphate | 0.282 | Vermiculite + Fe-P |
| Vermiculite | Iron phytate | 0.15 | Vermiculite + Fe-Phy |
| Vermiculite | Calcium monohydrogen phosphate | 0.218 | Vermiculite + Ca-P |
| Vermiculite | Calcium phytate | 0.148 | Vermiculite + Ca-Phy |
| Vermiculite | Calcium superphosphate | 0.514 | Vermiculite + Fertilizer-P |
|  |  |  |  |
| Experiment 3 | | | |
| Medium | Phosphate form | g pot^-1^ | P treatment |
| Vermiculite | No P applied | - | Vermiculite |
| Vermiculite | Aluminum phosphate | 0.515 | Vermiculite + Al-P |
| Vermiculite | Aluminum phytate | 0.484 | Vermiculite + Al-Phy |
| Vermiculite | Iron phosphate | 0.941 | Vermiculite + Fe-P |
| Vermiculite | Iron phytate | 0.5 | Vermiculite + Fe-Phy |
| Vermiculite | Calcium monohydrogen phosphate | 0.727 | Vermiculite + Ca-P |
| Vermiculite | Calcium phytate | 0.492 | Vermiculite + Ca-Phy |
| Vermiculite | Calcium superphosphate | 1.714 | Vermiculite + Fertilizer-P |

Table S2. Trace elements fertilizers solution

|  | The concentration of stock solution (mM) |
| --- | --- |
| EDTA-Fe | 53.7 mM |
| H_3_BO_3_ | 46.3 mM |
| MnCl_2_·_4_H_2_0 | 9.1 mM |
| ZnSO_4_·_7_H_2_0 | 0.77 mM |
| CuSO_4_·_5_H_2_0 | 0.32 mM |
| (NH_4_)_6_Mo_7_O_24_ | 0.03 mM |

Table S3. Transplanting plant P content and biomass

|  | Transplanting plant P content | Transplanting plant biomass |
| --- | --- | --- |
| Plants | mg plant^-1^ | g plant^-1^ |
| Guinea grass (Exp.1) | 0.02 | 0.04 |
| Guinea grass (Exp.2) | 0.019 | 0.0053 |
| Amaranth (Exp.1) | 0.03 | 0.032 |
| Chinese silver grass (Exp.1) | 0.02 | 0.036 |
| Chinese silver grass (Exp.2) | 0.017 | 0.0153 |
| Chinese silver grass (Exp.3) | 0.018 | 0.0178 |
| Sorghum (Exp.1) | 0.04 | 0.044 |
| Barley (Exp.1) | 0.03 | 0.049 |
| Potato (Exp.1) | 0.34 | 0.101 |
| Sunflower (Exp.1) | 0.39 | 0.103 |
| Radish (Exp.1) | 0.007 | 0.02 |
| Radish (Exp.2) | 0.069 | 0.0177 |
| Bahiagrass (Exp.1) | 0.002 | 0.004 |
| Bahiagrass (Exp.2) | 0.00176 | 0.0037 |
| Peanut (Exp.2) | 3.881 | 0.8208 |
| Soybean (Exp.1) | 1.24 | 0.263 |
| Soybean (Exp.2) | 1.74 | 0.544 |
